# Ibuprofen alters epoxide hydrolase activity and epoxy-oxylipin metabolites associated with different metabolic pathways in murine livers

**DOI:** 10.1038/s41598-021-86284-1

**Published:** 2021-03-29

**Authors:** Shuchita Tiwari, Jun Yang, Christophe Morisseau, Blythe Durbin-Johnson, Bruce D. Hammock, Aldrin V. Gomes

**Affiliations:** 1 Department of Neurobiology, Physiology, and Behavior, University of California, Davis, CA 95616 USA; 2 Department of Entomology and Nematology, and Comprehensive Cancer Center, University of California, Davis, CA 95616 USA; 3 Department of Public Health Sciences, University of California, Davis, CA USA; 4 Department of Physiology and Membrane Biology, University of California, Davis, CA USA

**Keywords:** Biochemistry, Lipidomics, Lipids, Metabolomics

## Abstract

Over the last decade oxylipins have become more recognized for their involvement in several diseases. Non-steroidal anti-inflammatory drugs (NSAIDs) such as ibuprofen are known to inhibit cyclooxygenase (COX) enzymes, but how NSAIDs affect oxylipins, in addition to COX products, in animal tissues is not well understood. Oxylipins in livers from male and female mice treated with 100 mg/kg/day of ibuprofen for 7 days were investigated. The results showed that ibuprofen treated male livers contained 7 times more altered oxylipins than ibuprofen treated female livers. In male and female livers some prostaglandins were altered, while diols, hydroxy fatty acids and epoxides were significantly altered in male livers. Some soluble epoxide hydrolase (sEH) products, such as 9,10-DiHODE were found to be decreased, while sEH substrates (such as 9(10)-EpODE and 5(6)-EpETrE) were found to be increased in male livers treated with ibuprofen, but not in ibuprofen treated female livers. The enzymatic activities of sEH and microsomal epoxide hydrolase (mEH) were elevated by ibuprofen in both males and females. Analyzing the influence of sex on the effect of ibuprofen on oxylipins and COX products showed that approximately 27% of oxylipins detected were influenced by sex. The results reveal that ibuprofen disturbs not only the COX pathway, but also the CYP450 and lipoxygenase pathways in male mice, suggesting that ibuprofen is likely to generate sex related differences in biologically active oxylipins. Increased sEH activity after ibuprofen treatment is likely to be one of the mechanisms by which the liver reduces the higher levels of EpODEs and EpETrEs.

## Introduction

Ibuprofen is the most common over-the-counter nonsteroidal anti-inflammatory drug (NSAID) known for its potent analgesic, anti-inflammatory, and antipyretic effects^1^. The therapeutic actions of ibuprofen are mediated by its ability to inhibit the synthesis and release of prostaglandins (PGs) E2 (PGE2) and I2 (PGI2) by blocking cyclooxygenase (COX) enzymes COX-1 and COX-2 which are involved in the generation of pain, fever, and inflammation^2,3^. COX-1 and COX-2 are the rate-determining enzymes for the synthesis of prostaglandins and other prostanoids including thromboxanes and prostacyclins^4^. These molecules play important roles in the immune, cardiovascular, gastrointestinal and renal system^3^. While COX-1 is important for the protection of gastric mucosa and platelet homeostasis through the formation of protective PGE2 and prostacyclin PGI2, COX-2 is involved with prostaglandin mediated pain and inflammation^4^.

Although NSAIDs are some of the most commonly used medicines worldwide, they are associated with various adverse side effects which are generally dose dependent^5^. The use of NSAIDs has been associated with gastrointestinal injury, hepatotoxicity, renal and cardiovascular disorders and hypertension^6,7^. The NSAIDs benoxaprofen and bromfenac were withdrawn from the market due to severe hepatic toxicity^8,9^. Similarly, higher incidence of myocardial infarction and stroke led to the withdrawal of rofecoxib and valdecoxib (Bextra)^10^. In addition, the NSAIDs nimesulide, diclofenac and sudoxicam were reported to cause acute liver injury^11,12^. In April 2019, The French National Agency for the Safety of Medicines and Health Products (ANSM) issued a warning regarding the use of NSAIDs (ibuprofen and ketoprofen) for the treatment of patients with infectious diseases^13,14^.

Another concern about ibuprofen is its presence in the environment. Data suggests that fish health could be adversely affected by the levels of ibuprofen found in rivers^15,16^. Ibuprofen is also found in drinking water and surface wells. Concerns exist about its possible biological effects when taken regularly in small amounts^17,18^. Limited studies have investigated the effect of NSAIDs on oxylipins in the P450 branch of the arachidonic acid cascade and these studies largely utilized blood samples. In general, oxylipins regulate a variety of physiological functions by acting as important signaling molecules for specific receptors or by modulating transcription factors and ion channels in an autocrine manner^19^. Perturbations in the P450 generated oxylipin concentrations have been shown to be associated with various pathological conditions including chronic pain^20,21^, diabetes^22^, Alzheimer’s disease^23,24^, and cardiovascular diseases^25^. Oxylipins are bioactive oxidized fatty acid metabolites synthesized by the oxidation of polyunsaturated fatty acids (PUFAs).

In a placebo-controlled phase III selenium/celecoxib trial to prevent colorectal adenomatous polyps, blood oxylipins from celecoxib treated participants showed higher levels of individual CYP450 and LOX metabolites and lower levels of COX-derived metabolites compared to placebo treated participants^26^. However, since circulating oxylipin profiles are unlikely to represent tissue profiles, and as we have previously shown that ibuprofen was associated with proteasome and metabolic dysfunction in livers^27^, the oxylipin profiles of livers from ibuprofen treated mice were investigated.

We hypothesized that ibuprofen would significantly reduce the prostaglandins and thromboxanes that are produced by COX1 and COX2 and would have few effects on non-COX related arachidonic acid pathways. Our data suggest that a moderate dose of ibuprofen treatment for 7 days significantly altered oxylipins related to different metabolic pathways and revealed significant sex specific differences in livers from ibuprofen treated males and females relative to their vehicle controls.

## Materials and methods

### Animal studies

C57BL/6 J male and female mice (8-week age) were used for the study. The animal experiment was performed in accordance with the protocols approved by the Institutional Animal Care and Use Committee (IACUC) of the University of California, Davis. This study is reported in accordance with the ARRIVE guidelines (https://arriveguidelines.org). Mice were maintained at controlled temperature and humidity and had free access to food and water. Mice were treated with 100 mg/kg/day of ibuprofen for 7 days in drinking water. The dose of ibuprofen given to mice would be approximately equivalent to a human taking 486 mg/day as previously described^27^. This dose of ibuprofen is significantly lower than the maximum suggested over-the-counter dose (800–1200 mg/day). The mice were sacrificed, and livers were collected and quickly washed twice in ice-cold phosphate buffered saline (PBS). The tissues were then pulverized in liquid nitrogen, collected in clean microcentrifuge tubes, and stored at − 80 °C until needed.

### LC–MS/MS-based lipidomic analysis

Extraction and analyses of the regulatory lipid mediator: The extraction process is similar to the protocol as described in a previously published paper^28^. 10 µL of methanol containing deuterated internal standard solution, a mixture of d4 PGF1α, d4 TXB2, d4 PGE2, d4 LTB4, d11 14,15 DiHETrE, d6 20 HETE, d4 9 HODE, d8 5 HETE, d11 11,12 EpETrE (Cayman Chemical, Ann Arbor, MI), was added to approximately 100 mg of liver tissue. 400 µL of cold methanol containing 0.1% acetic acid and 0.1% butylated hydroxytoluene, BHT (Sigma-Aldrich, St. Louis, MO) solution was added to these tissue samples and stored at − 80 °C for 30 min. After freezing, samples were homogenized using Retsch MM301 ball mills (Retsch Gmbh, Germany) at 30 Hz for 10 min and then kept at − 80 °C overnight. The homogenates were centrifuged at 16,000 g for 10 min, the supernatants were collected, and remaining pellets were washed with 100 µL of ice-cold methanol with 0.1% acetic acid and 0.1% BHT and centrifuged at 16,000 g for 10 min. The supernatants of each sample were combined and diluted with 2 mL of H_2_O and loaded onto Waters Oasis HLB 3 cc (Waters, Milford, MA) solid phase extraction (SPE) cartridges.

The oxylipins were measured on a 1200 SL ultra-high-performance liquid chromatography (UHPLC) (Agilent, Santa Clara, CA) interfaced with a 4000 QTRAP mass spectrometer (Sciex, Redwood City, CA). The separation conditions for LC were optimized to separate the critical pairs of oxylipins, which share the same multiple reaction monitor (MRM) transitions. In brief, separation was achieved on an Agilent Eclipse Plus C18 150 × 2.1 mm 1.8 um column with mobile phases of water with 0.1% acetic acid as mobile phase A and acetonitrile/methanol (84/16) with 0.1% acetic acid as mobile phase B. All the parameters on the mass spectrometer were optimized with pure standards (purchased from Cayman Chemical, Ann Arbor, MI) under negative mode^28,29^. A scheduled multiple reaction monitoring (MRM) scan mode was employed to increase the sensitivity of the measurement. The analytical variance and lower limit of quantification for oxylipins are shown in the supplementary data.

### LC–MS/MS-based proteomic analysis

Proteomic analysis was carried out as previously described and used the same data as published in Shuchita et al. 2020^27^. Scaffold version 4.10 was used to analyze, quantify, and display the primary amino acid sequence of the sEH and mEH (Proteome Software Inc. Oregon).

### sEH and mEH activity assay

Pulverized liver samples were weighed (50 mg) and homogenized in ice cold 1X PBS buffer containing 0.1% ethylenediaminetetraacetic acid (EDTA), protease inhibitor (PMSF 1 mM) and 1 mM DTT. The supernatant was separated and quantified by the bicinchoninic acid (BCA) method using bovine serum albumin (BSA) as standard. The samples were flash frozen and sEH activity was measured as reported previously using [3H]-t-DPPO as substrate with [S] = 50 μM and incubation for 10 to 30 min at 30 °C^30^.

#### Activity measurement

##### sEH-H like activity

To measure the residual soluble epoxide hydrolase (sEH-H) activity, [3H]-trans-diphenyl-propene oxide (t-DPPO) was used as substrate^31^. Briefly, 1 µL of a 5 mM solution of t-DPPO in DMSO was added to 100 µL of the tissue extracts diluted in sodium phosphate buffer (100 mM, pH 7.4) containing 0.1 mg/mL BSA ([S]_final_ = 50 µM). The mixture was incubated at 37 °C for 5–20 min, and the reaction was quenched by addition of 60 µL of methanol and 200 µL of isooctane, which extracts the remaining epoxide from the aqueous phase. Extractions of an identical reaction with 1-hexanol were performed in parallel to assess the possible presence of glutathione transferase activity which could also transform the substrate^31^. Hexanol extracts epoxides and diols into the hyperphase while glutathione conjugates remain in the hypophase. The activity was followed by measuring the quantity of radioactive diol formed in the aqueous phase using a scintillation counter (TriCarb 2810 TR, Perkin Elmer, Shelton, CT). Assays were performed in triplicate.

mEH like activity: The presence of microsomal epoxide hydrolase (mEH) activity was determined using [3H]-cis-stilbene oxide (c-SO) as substrate^32^. The tissue extracts were diluted in Tris/HCl buffer (0.1 M, pH 9.0) containing 0.1 mg/mL BSA. The reaction was started by adding 1 µL of a 5 mM solution of c-SO in ethanol to 100 µL of diluted extracts([S]_final_ = 50 µM). The mixture was incubated at 37 °C for 5–30 min, and then at 30 °C before substrate introduction. The reaction was then quenched by adding 250 μL of isooctane, which extracts the remaining epoxide from the aqueous phase. Extractions with 1-hexanol were performed in parallel to assess the possible presence of glutathione transferase activity which could also transform the substrate REF 1. The activity was followed by measuring the quantity of radioactive diol formed in the aqueous phase using a scintillation counter. Assays were performed in triplicate.

### Western blotting

#### Sample preparation

Liver tissues were pulverized in liquid nitrogen and 20 mg were weighed and homogenized in ice cold 1X RIPA buffer (50 mM Tris, 150 mM sodium chloride (NaCl), 1% NP40, 0.5% sodium deoxycholate and 0.1% SD, pH 8) using a glass Dounce homogenizer. The supernatants were separated after centrifugation at 12,000 g for 15 min at 4 °C. The quantification of the proteins was done using the BCA Protein Assay (Bio-Rad, Cat. #500–0119) and the samples were further diluted to equal protein concentrations. Liver samples were then mixed with 4X Laemmli sample buffer (8% SDS, 40% glycerol, 0.4% bromophenol blue, 240 mM Tris, pH 6.8). The β-mercaptoethanol was added freshly and samples were then boiled for 5 min at 95 °C.

#### Electrophoresis and western blotting

4–20% 18-well TGX precast gels (Cat. # 567–1094, Bio-Rad) were used to separate 20 µg of protein in each lane. The proteins on the TGX precast gel were then transferred to a nitrocellulose membrane (Trans-Blot Turbo Midi Nitrocellulose, #170–4159, Bio-Rad) using the Bio-Rad Trans-Blot Turbo Transfer System (Cat. # 170–4155, Bio-Rad). After staining the membranes with Ponceau S they were imaged and used as a loading control for total protein normalization of Western blots. 3% nonfat dry milk (NFM) (Cat. # 170–6404, Bio-Rad) in Tris-buffered saline (TBS) (50 mM Tris, 150 mM NaCl, pH 7.5) containing 0.05% (wt/vol) Tween 20 (TTBS) was used to block empty sites on the membranes for 1 h at room temperature. The membranes were then incubated in 1% TTBS with the primary antibodies diluted to 1:5000 for mouse anti-sEH^33^ or 1:2000 for mouse anti-mEH^34^ and left overnight at 4 °C with gentle shaking. Thereafter, membranes were washed in TTBS three times for 5 min each**,** incubated with horseradish peroxidase conjugated rabbit anti-mouse IgG secondary antibody (Sigma-Aldrich, anti-mouse Cat. # A9044) diluted 1:10,000 in 1% TTBS for 1 h at room temperature and washed three more times in 1X TTBS for 5 min each with gentle shaking. Blots were then developed using enhanced commercial chemiluminescent reagent (Clarity, Bio-Rad, 170–5061) and were imaged in ChemiDoc MP (Bio-Rad). Image Lab 5.0 was used to quantify the blots.

#### Data analysis

Results in Table 1 are expressed as means ± standard deviations (SD) from at least four independent experiments. To determine the differences between control and ibuprofen treated groups and male versus female differences the raw data were first log2 transformed. The data was then normalized using cyclic loess normalization^35^, as implemented in the Bioconductor package limma^36^, version 3.44.3. Limma was initially developed for microarray data, but the fitting routines of this program are not limited to microarrays. Differential oxylipin abundance analyses were conducted using limma, which fits a linear model to data from each lipid using empirical Bayes smoothing to improve estimates of standard errors of log fold changes^37^, and calculates Benjamini-Hochberg (BH) false-discovery rate p-values for each lipid^38^. The BH procedure decreases the false discovery rate and helps avoid false positives. The linear model used in limma for this analysis was a two-factor ANOVA model including effects for group, sex, and their interaction. Analyses were conducted using R version 4.0.2 (2020-06-22)^39^. Adjusted p-values of < 0.05 were defined as statistically significant.

**Table 1:** Levels of oxylipins in liver tissue of mice.

Male			Female		
Oxylipin	Control (n = 13)	IB (n = 15)	Oxylipin	Control (n = 12)	IB (n = 14)
**PG & Others**					
6-keto-PGF1a	138 ± 89.0	2.60 ± 2.55*	6-keto-PGF1a	184 ± 99.0	6.12 ± 4.53*
TXB2	29.8 ± 13.2	3.87 ± 0.69*	TXB2	39.5 ± 26.8	5.82 ± 3.77*
PGF2a	52.1 ± 23.2	1.06 ± 0.43*	PGF2a	59.3 ± 45.3	1.84 ± 0.90*
PGE2	5.76 ± 8.81	0.09 ± 0.15*	PGE2	6.00 ± 3.43	0.69 ± 0.34*
PGD2	10.9 ± 6.5	0.78 ± 0.31*	PGD2	12.1 ± 7.87	1.01 ± 0.41*
5-oxo-ETE	3.95 ± 2.12	4.43 ± 2.11	5-oxo-ETE	73.7 ± 64.2	160 ± 366
12-oxo-ETE	39.7 ± 36.1	50.3 ± 20.0	12-oxo-ETE	395 ± 258	596 ± 1011
15-oxo-ETE	6.30 ± 5.28	6.16 ± 1.96	15-oxo-ETE	30.1 ± 19.9	27.2 ± 12.9
LXA4	1.80 ± 0.70	1.80 ± 0.45	LXA4	3.52 ± 2.40	3.43 ± 2.42
15-deoxy-PGJ2	37.9 ± 12.9	39.2 ± 11.1	15-deoxy-PGJ2	29.6 ± 11.6	30.5 ± 14.2
**Diols**					
9,10-DiHOME	37.8 ± 9.39	21.8 ± 54.9*	9,10-DiHOME	35.6 ± 27.3	27.7 ± 6.53
12,13-DiHOME	171 ± 242	96.6 ± 33.3*	12,13-DiHOME	155 ± 72.4	128 ± 30.8
9,10-DiHODE	3.88 ± 8.64	1.11 ± 0.46*	9,10-DiHODE	1.98 ± 1.10	1.46 ± 0.40
12,13-DiHODE	7.60 ± 20.0	1.63 ± 0.53*	12,13-DiHODE	3.45 ± 2.00	2.45 ± 0.81
15,16-DiHODE	49.6 ± 97.1	18.8 ± 6.56*	15,16-DiHODE	50.4 ± 22.2	44.5 ± 23.2
5,6-DiHETrE	3.02 ± 1.61	3.20 ± 1.23	5,6-DiHETrE	2.85 ± 1.22	2.95 ± 1.09
8,9-DiHETrE	6.59 ± 3.95	6.54 ± 2.13	8,9-DiHETrE	12.3 ± 5.29	14.4 ± 3.86
11,12-DiHETrE	14.3 ± 6.62	14.1 ± 6.38	11,12-DiHETrE	22.8 ± 10.17	27.9 ± 7.93
14,15-DiHETrE	42.2 ± 15.3	43.8 ± 17.2	14,15-DiHETrE	49.9 ± 26.41	55.2 ± 17.6
8,15-DiHETE	8.76 ± 4.37	10.2 ± 3.85	8,15-DiHETE	15.0 ± 10.76	14.4 ± 6.23
11,12-DiHETE	1.47 ± 0.56	1.72 ± 0.62	11,12-DiHETE	1.86 ± 0.67	2.35 ± 1.22
14,15-DiHETE	4.27 ± 1.28	5.20 ± 1.95	14,15-DiHETE	4.87 ± 2.16	5.60 ± 2.09
17,18-DiHETE	10.3 ± 1.95	10.8 ± 3.63	17,18-DiHETE	12.5 ± 4.39	14.5 ± 5.23
4,5-DiHDPE	28.0 ± 24.9	25.5 ± 12.3	4,5-DiHDPE	13.89 ± 13.69	5.28 ± 3.98
7,8-DiHDPE	ND	ND	7,8-DiHDPE	2.02 ± 0.84	2.23 ± 0.39
10,11-DiHDPE	2.94 ± 1.26	3.15 ± 1.06	10,11-DiHDPE	4.68 ± 2.05	5.41 ± 1.97
13,14-DiHDPE	4.61 ± 1.60	5.17 ± 2.38	13,14-DiHDPE	7.67 ± 3.47	8.30 ± 3.56
16,17-DiHDPE	13.8 ± 4.10	14.8 ± 6.18	16,17-DiHDPE	16.9 ± 8.40	16.7 ± 4.95
19,20-DiHDPE	55.2 ± 11.7	55.6 ± 15.7	19,20-DiHDPE	62.8 ± 23.5	70.8 ± 23.0
**Hydroxy Fatty acids**					
9-HODE	1047 ± 605	623 ± 220*	9-HODE	992 ± 647	907 ± 455
13-HODE	1415 ± 810	1046 ± 385	13-HODE	1743 ± 1139	1445 ± 450
9-HOTrE	20.1 ± 30.3	9.76 ± 2.93*	9-HOTrE	27.2 ± 34.9	23.6 ± 16.7
13-HOTrE	18.7 ± 27.5	9.07 ± 4.24*	13-HOTrE	39.6 ± 43.5	47.2 ± 49.4
15-HETrE	74.3 ± 34.8	113 ± 54.8	15-HETrE	213 ± 181	569 ± 1373
5-HETE	30.2 ± 21.2	30.7 ± 7.8	5-HETE	89.26 ± 74.3	103.1 ± 89.6
8-HETE	28.5 ± 16.1	32.9 ± 11.0	8-HETE	269 ± 389	429 ± 1013
9-HETE	29.7 ± 17.2	34.2 ± 10.9	9-HETE	154 ± 191	131 ± 167
11-HETE	114 ± 43.0	97.1 ± 34.0*	11-HETE	501 ± 424	410 ± 542
12-HETE	283 ± 113	380 ± 195	12-HETE	2014 ± 1197	3319 ± 7329
15-HETE	252 ± 117	291 ± 99.0	15-HETE	1036 ± 811	976 ± 1309
20-HETE	3.07 ± 1.5	3.14 ± 1.7	20-HETE	1.79 ± 0.9	2.02 ± 0.84
5-HEPE	6.21 ± 5.1	5.59 ± 1.9	5-HEPE	18.1 ± 12.8	12.1 ± 5.1
8-HEPE	6.84 ± 5.2	28.5 ± 35.7*	8-HEPE	14.4 ± 17.7	17.1 ± 15.6
12-HEPE	125 ± 113	668 ± 592*	12-HEPE	217 ± 27	221 ± 500
15-HEPE	7.01 ± 3.1	5.95 ± 3.0	15-HEPE	48.7 ± 54.2	126 ± 301
17-HDoHE	56.4 ± 34.8	55.3 ± 25.5	17-HDoHE	289.82 ± 389.9	770 ± 2186
**Epoxides**					
9(10)-EpOME	78.3 ± 79.1	188 ± 164	9(10)-EpOME	88.19 ± 99.0	65.5 ± 38.4
12(13)-EpOME	73.0 ± 68.5	178.3 ± 164*	12(13)-EpOME	67.06 ± 65.7	59.4 ± 40.1
9-HOTrE	20.1 ± 30.3	9.76 ± 2.9*	9-HOTrE	27.18 ± 34.9	23.8 ± 16.7
5(6)-EpETrE	301 ± 249	954 ± 931*	5(6)-EpETrE	305.5 ± 340	225 ± 148
8(9)-EpETrE	14.6 ± 12.5	49.9 ± 47.6*	8(9)-EpETrE	28.2 ± 27.4	25.0 ± 9.0
11(12)-EpETrE	30.0 ± 23.7	112 ± 111*	11(12)-EpETrE	48.7 ± 54.3	42.2 ± 19.6
14(15)-EpETrE	27.0 ± 23.2	103 ± 93.3*	14(15)-EpETrE	41.8 ± 60.4	30.1 ± 16.2
9(10)-EpODE	6.37 ± 6.9	15.4 ± 13.5*	9(10)-EpODE	7.24 ± 8.3	4.59 ± 3.3
12(13)-EpODE	3.79 ± 4.1	8.97 ± 7.6*	12(13)-EpODE	4.48 ± 4.9	3.03 ± 2.0
15(16)-EpODE	18.3 ± 31.1	28.4 ± 23.2*	15(16)-EpODE	23.3 ± 20.6	12.5 ± 8.5
8(9)-EpETE	17.8 ± 22.8	139 ± 201*	8(9)-EpETE	30.7 ± 19.9	23.8 ± 17.0
11(12)-EpETE	1.21 ± 1.1	4.94 ± 4.4*	11(12)-EpETE	ND	ND
14(15)-EpETE	2.00 ± 1.9	8.95 ± 9.4*	14(15)-EpETE	3.01 ± 3.3	2.09 ± 1.5
17(18)-EpETE	2.52 ± 2.0	10.8 ± 10.3*	17(18)-EpETE	3.61 ± 3.9	2.56 ± 1.9
7(8)-EpDPE	135 ± 94.2	392 ± 306*	7(8)-EpDPE	226 ± 189	185 ± 96.0
10(11)-EpDPE	10.9 ± 9.4	30.1 ± 23.3*	10(11)-EpDPE	14.1 ± 12.7	11.7 ± 5.6
13(14)-EpDPE	7.43 ± 6.4	22.1 ± 16.5*	13(14)-EpDPE	9.00 ± 8.6	7.6 ± 3.6
16(17)-EpDPE	8.35 ± 7.4	23.8 ± 17.9*	16(17)-EpDPE	9.94 ± 10.5	8.00 ± 4.0
19(20)-EpDPE	12.6 ± 9.2	33.7 ± 23.2*	19(20)-EpDPE	19.3 ± 15.0	14.00 ± 8.9
**Keto fatty acids**					
EKODE	11.2 ± 13.4	8.5 ± 2.7	EKODE	37.6 ± 18.4	29.9 ± 25.1
9-oxo-ODE	57.0 ± 51.3	50.7 ± 14.6*	9-oxo-ODE	205 ± 179	139 ± 84.5
13-oxo-ODE	ND	ND	13-oxo-ODE	6.04 ± 5.7	5.18 ± 3.5
**Trihydroxy Fatty acids**					
9,12,13-TriHOME	ND	ND	9,12,13-TriHOME	2302 ± 2213	2077 ± 3612
9,10,13-TriHOME	ND	ND	9,10,13-TriHOME	1231 ± 1128	1139 ± 1880

ND, not detected. Results are in ng/mL, mean ± standard deviation (mean ± SD). Significance level for the comparison between control and ibuprofen treated groups using the procedures described in the methods section. The metabolites are arranged in each section by chain length, then double bond so that LA, ALA, AA, EPA, and DHA metabolites (if present) are separated. * Adjusted P value < 0.05.

## Results

**Figure 1:**
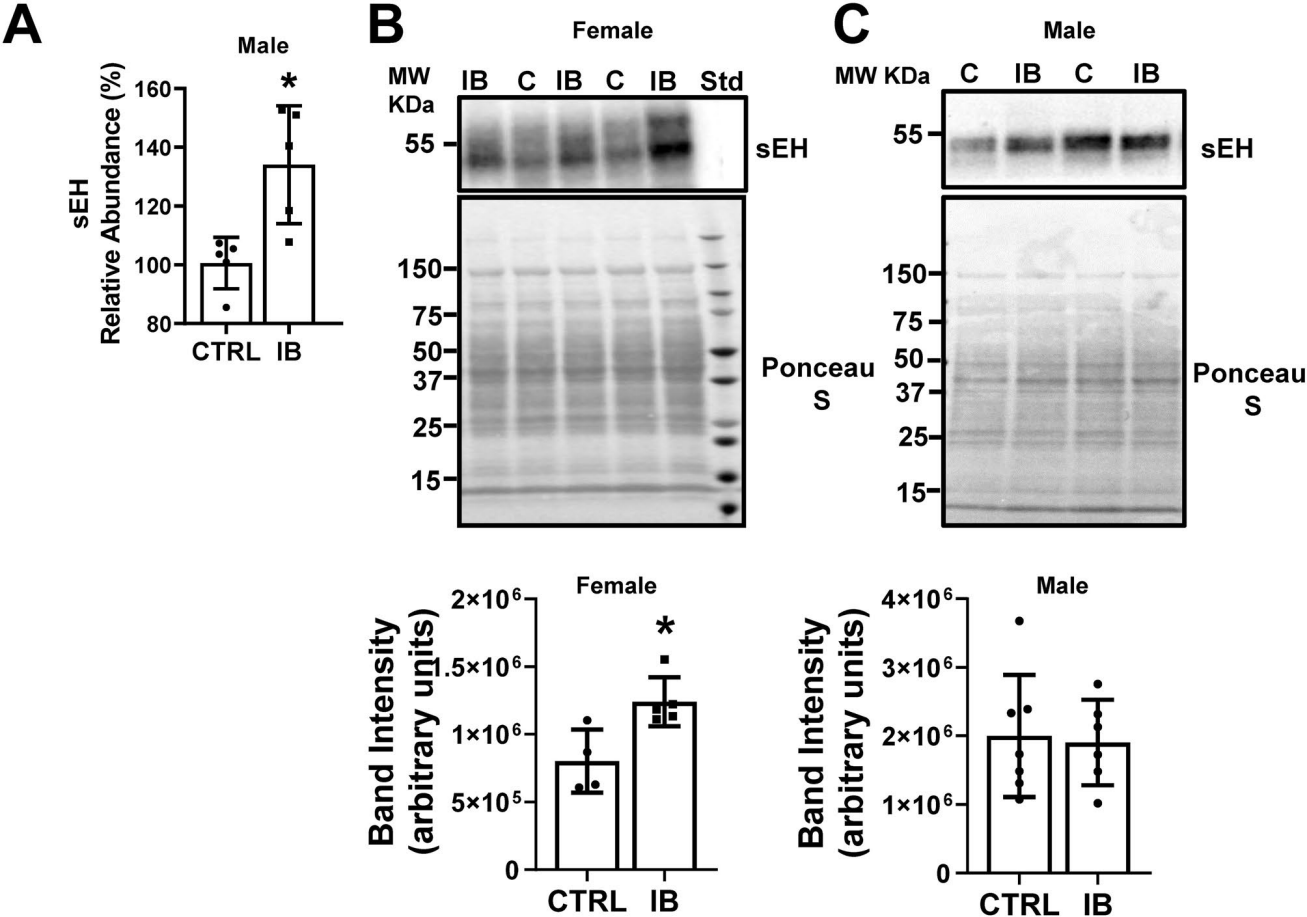
Characterization of sEH expression in control and ibuprofen treated mouse livers. (**A**) Abundance of sEH in ibuprofen treated livers relative to control as determined by TMT mass spectrometry (n = 5), (**B**) Relative expression of sEH in livers from female mice as determined by Western blotting (n = 6), (**C**) Relative expression of sEH in livers from male mice as determined by Western blotting (n = 6). (**B**) and (**C**) are cropped images from different blots. IB, ibuprofen treated liver samples, (**C**), control liver samples. Values are mean ± SE; *p < 0.05. Full length blots are included in the Supplementary Information file.

**Figure 2:**
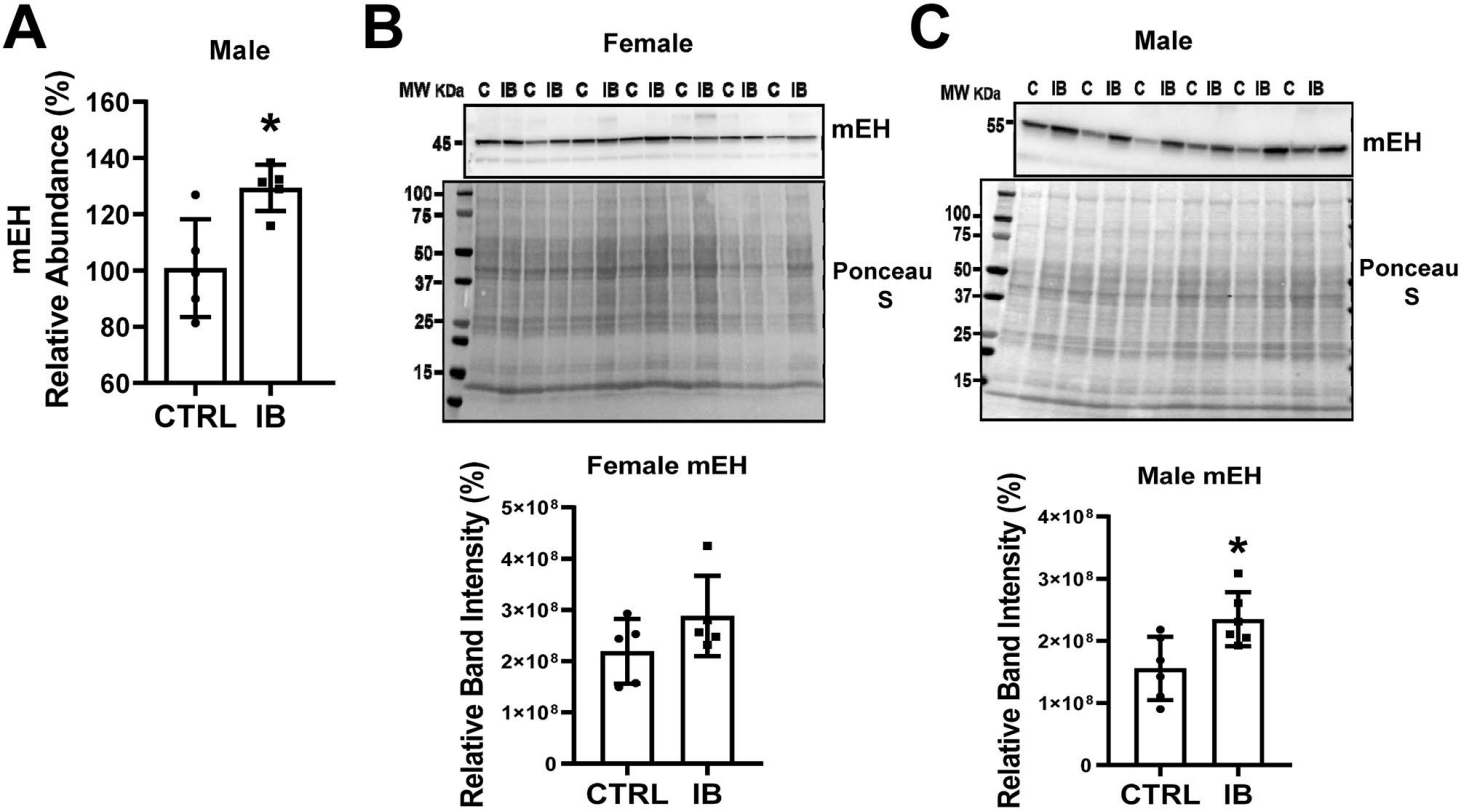
Characterization of mEH expression in control and ibuprofen treated mouse livers. (**A**) Abundance of mEH as determined by TMT mass spectrometry (n = 5), (**B**) Relative expression of mEH in livers from female mice as determined by Western blotting (n = 6), (**C**) Relative expression of mEH in livers from male mice as determined by Western blotting (n = 6). (**B**) and (**C**) are cropped images from different blots. Full length blots are included in the Supplementary Information file. IB, ibuprofen treated liver samples, (**C**), control liver samples. Values are mean ± SE; *p < 0.05.

The livers from male and female mice treated with ibuprofen or vehicle were analyzed by mass spectrometry. Oxylipin profiles of ibuprofen treated mice showed that 35 oxylipins in male livers and 5 oxylipins in female livers were significantly altered compared to livers from vehicle treated male and female mice respectively (Table 1). Substrates of sEH were increased in livers from ibuprofen treated male mice (Table 1). To determine if the increased sEH substrate levels may be due to changes in the protein levels of sEH the Scaffold program was used to extract sEH information from previously analyzed data^27^. In these earlier studies, livers from 5 vehicle control and 5 ibuprofen treated male mice were used for Tandem Mass Tag (TMT) proteomics. The proteomic experiments were only carried out on male mice. The mass spectrometry results suggest that both sEH and mEH were significantly upregulated in livers of male mice treated with ibuprofen (Figs. 1A and 2A). A representative spectrum of sEH, the intensity of the TMTs for that peptide and the amino acid residues that were detected by mass spectrometry are shown in supplemental Fig. 1. A similar representative spectrum for mEH, the TMT intensities for that peptide, and the mEH residues detected by mass spectrometry are shown in supplemental Fig. 2. As far we know, this is the first report of sEH and mEH being upregulated by ibuprofen treatment.

Validation of the mass spectrometry data using Western blotting showed that sEH was upregulated in livers from females treated with ibuprofen (Fig. 1B). However, no change in the expression level of sEH was observed in ibuprofen treated male livers compared to normal control livers (Fig. 1C). The livers used for the Western blotting were different from the livers used for mass spectrometry and may show intrinsic variability between mice. Validation of the mass spectrometry data for mEH using Western blotting showed that the livers from ibuprofen treated female mice had elevated mEH expression, but not statistically significant higher levels when compared to controls (Fig. 2B). The male mice treated with ibuprofen had higher levels of liver mEH than controls (Fig. 2C), consistent with the proteomic data.

### Effect of ibuprofen on sEH and mEH activities in mice liver

**Figure 3:**
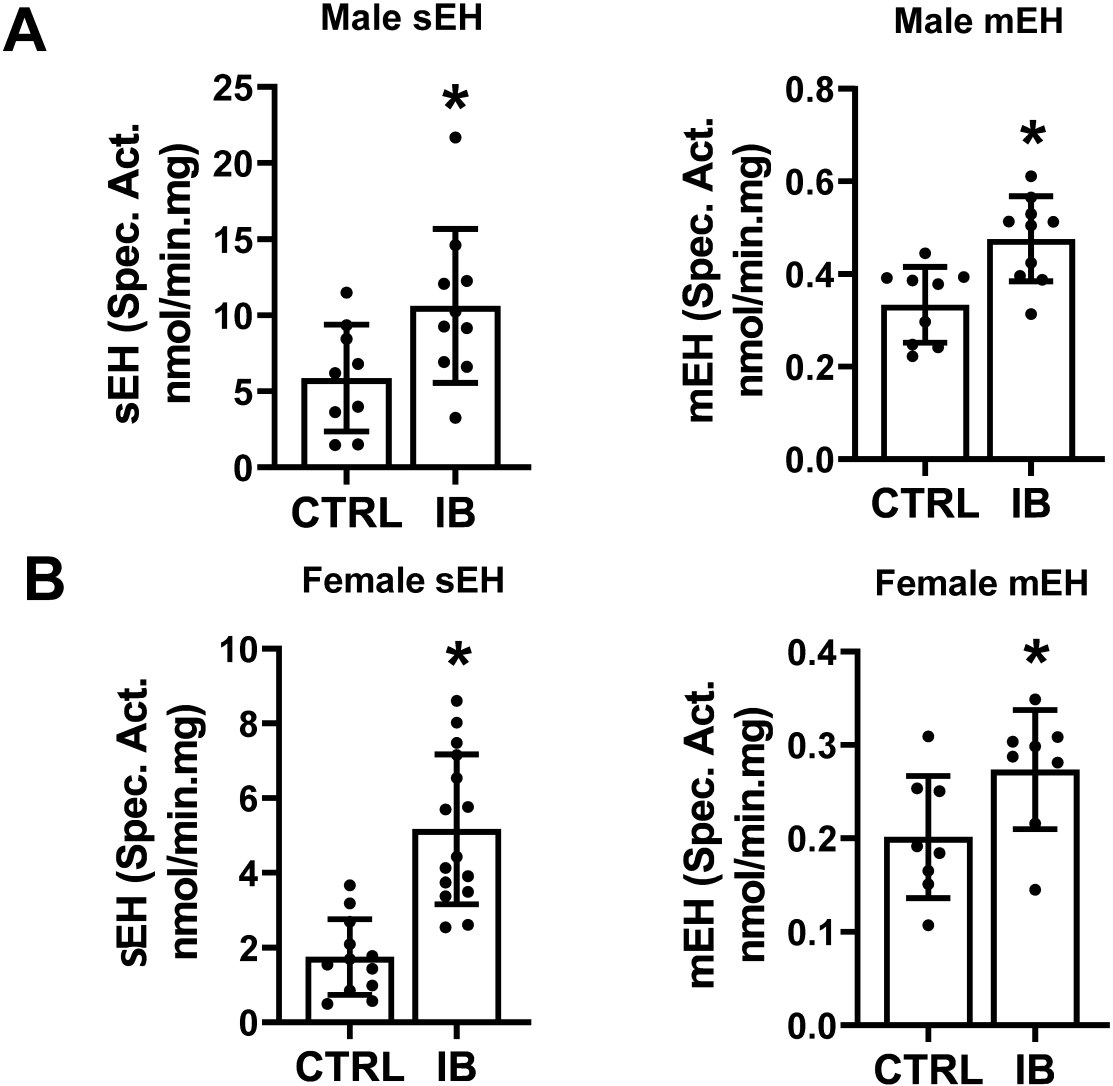
Characterization of sEH and mEH activities in control and ibuprofen treated murine livers. (**A**) sEH activity and mEH activity in male mouse liver. (**B**) sEH activity and mEH activity of female liver lysates. Values are mean ± SE; n = 12–15 per group. *p < 0.05.

sEH is a promising target for the treatment of hypertension, inflammatory diseases, pain, diabetes, and stroke^40–43^. Since the proteomic results suggest that sEH and mEH may not be elevated under some conditions of ibuprofen treatment, we decided to investigate if ibuprofen altered sEH and mEH activities. Using larger numbers of liver samples, the activities of sEH and mEH in ibuprofen treated samples were found to be statistically increased in both males and females (Fig. 3). These results suggest that moderate ibuprofen treatment to mice significantly increased sEH and mEH activities in both male and female livers relative to their controls.

### CYP-derived oxylipins

**Figure 4:**
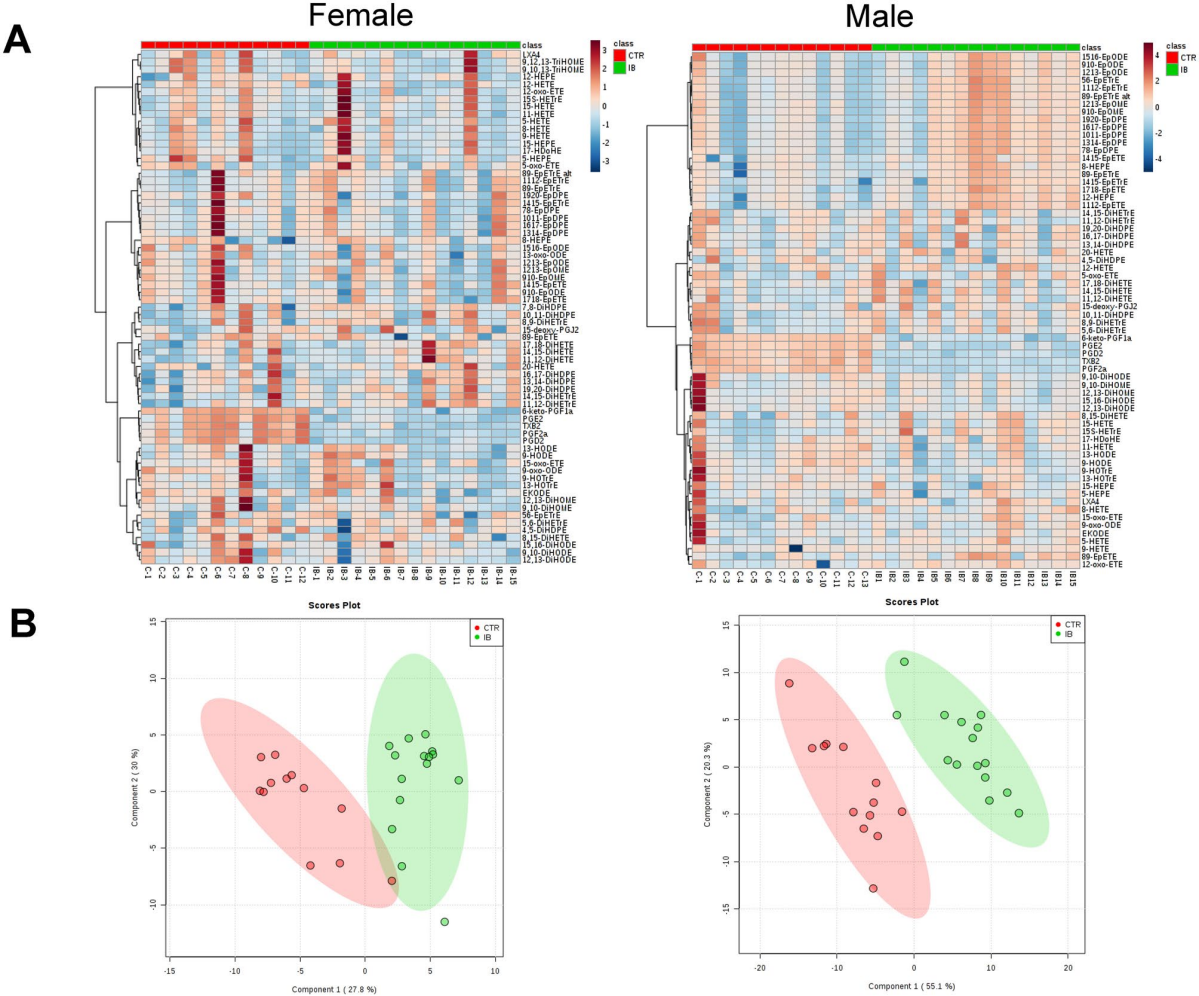
Short-term ibuprofen treatment (7 days) altered oxylipin profiles in murine liver. (**A**) Heatmap showing the eicosanoid profile shifted by ibuprofen in control and ibuprofen treated groups in female and male mice liver. The relative intensities of variables in control and ibuprofen treated livers were shown by color bars. (**B**) Partial least squares-discriminant analysis** (**PLS-A**) **score plots of female and male mice livers (n = 12–15 per group).

Heat maps of the oxylipin changes observed in male and female mice are shown in Fig. 4A. Partial least squares-discriminant analysis** (**PLS-DA**) **score plots of male and female mice liver oxylipin data suggest that males and females respond very differently to ibuprofen treatment (Fig. 4B). PLS-DA is utilized to show discrimination between the control and ibuprofen treated samples. Among the three major oxylipin metabolizing pathways (COX, LOX, and CYP), COX derived components showed the largest decreases in amounts while the CYP-derived oxylipins showed the most sex specific changes in ibuprofen treated mice livers relative to their normal controls (Table 1). In total, 69 different oxylipins were detected in quantifiable amounts in the mouse liver samples.

#### Effect of ibuprofen on CYP-derived oxylipins through the AA pathway

The CYP pathway utilizes AA as a substrate to produce oxylipins by the epoxygenase and ω-hydroxylase pathways. Epoxy fatty acids (EpFA) are produced in response to vascular endothelial inflammation and are anti-inflammatory and vasodilators in function^44,45^. The data suggest that ibuprofen altered CYP-derived EpFA oxylipins, including 5(6)-EpETrE, 8(9)-EpETrE, 11(12)-EpETrE and 14(15)-EpETrE. These oxylipins were significantly elevated in ibuprofen treated male livers relative to their controls (Table 1. Supplementary Fig. 3). No statistically significant differences in the levels of CYP-derived oxylipins were observed between the control and ibuprofen treated female groups, suggesting sex specific differences (Table 1).

#### Effect of ibuprofen on CYP-derived oxylipins through the EPA pathway

**Figure 5:**
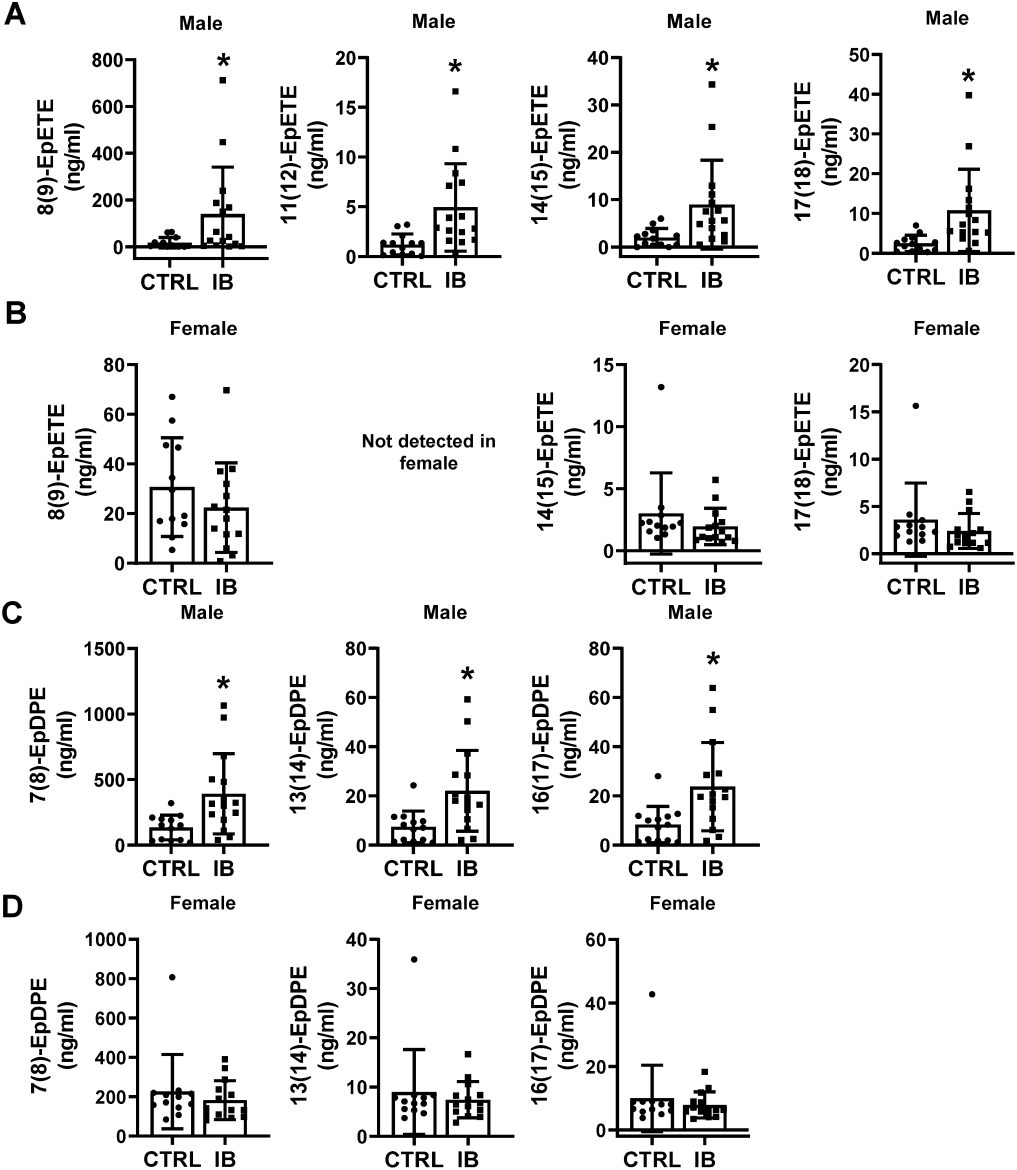
Schematic diagram showing CYP450 derived oxylipins from EPA and DHA which are altered by ibuprofen treatment. Boxplots showing the change in the levels of CYP-derived oxylipins from EPA in (**A**) male liver and (**B**) female liver, and CYP-derived oxylipins from DHA in male (**C**) and female (**D**) liver. Values are mean ± SE; n = 12–15 per group. *p < 0.05.

Similar to AA, EPA acts as a substrate for CYP enzymes to produce fatty acid epoxides (EpETEs) which are further metabolized to dihydroxy fatty acids (DiHETEs) by sEH^31^. The ω- hydroxylation of EPA yield HEPEs such as 10-HEPE, 19-HEPE and 20-HEPE^19,31^. Our analysis found that CYP derived oxylipins from the EPA pathway such as 8-HEPE, 12-HEPE, 8(9)-EpETE, 11(12)-EpETE, 14(15)-EpETE, and 17,18 EpETE were significantly increased in male livers treated with ibuprofen compared to their control counterparts (Table 1, Fig. 5A). Interestingly, 8(9)-EpETE and 17(18)-EpETE in ibuprofen treated female livers trended toward a decrease relative to controls (Table 1, Fig. 5B). No difference was observed in levels of EPA derived oxylipins such as 5-HEPE, 15-HEPE, 8,9-DiHETE, 11,12-DiHETE, 14,15-DiHETE and 17,18-DiHETE in both male and female livers from ibuprofen treated mice relative to controls (Table 1).

#### Effect of ibuprofen on CYP-derived oxylipins through DHA pathway

The fatty acid epoxides obtained from DHA via CYP enzymes generate epoxides such as 4,5-EpDPE, 7,8-EpDPE, and 16,17-EpDPE^19,46,47^. These epoxides can be further metabolized by sEH to diols such as 16,17-dihydroxy-docosapentaenoic acid (16,17 DiHDPE)^31^. Similarly, ω-hydroxylase activity of CYP produces HDoHE from DHA with hydroxyl groups near the methyl end of DHA^48^. Similar to EpETE, CYP-derived EpFA from EPA and DHA have anti-inflammatory, vasodilatory, and anticancer effects^49,50^. The levels of 7(8)-EpDPE, 11(12)-EpDPE, 13(14)-EpDPE, 16(17)-EpDPE, and 19(20)-EpDPE were all increased in ibuprofen treated male livers but not in livers form ibuprofen treated female mice (all relative to their respective controls) (Fig. 5C and D). No change was observed in the levels of DiHDPEs in livers from either male or female mice treated with ibuprofen compared to controls (Table 1).

#### Effect of ibuprofen on CYP-derived oxylipins through α-linoleic acid (ALA) pathway

**Figure 6:**
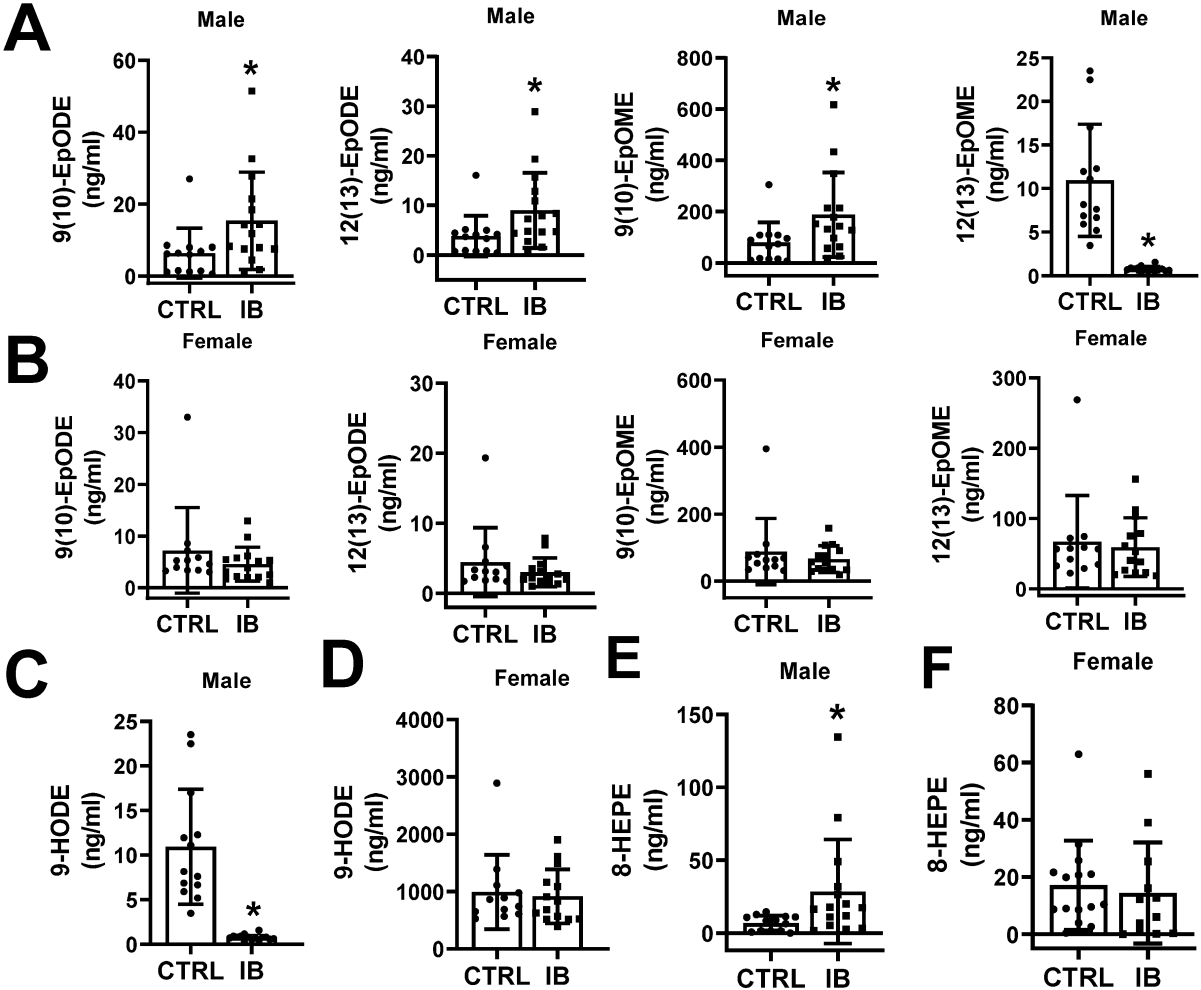
Effect of ibuprofen treatment on CYTP450 derived oxylipins from ALA, LA, and EPA in liver tissue of mice. Boxplots showing the change in the levels of CYP-derived oxylipins from ALA in (**A**) male liver and (**B**) female liver, LA (**C**) male and (**D**) female liver, and EPA-derived oxylipins in male (**E**) and female (**F**) liver. Values are mean ± SE; n = 12–15 per group. *p < 0.05.

αLA via CYP P450 activity produces EpFA such as 12,13-EpODE which can be further metabolized to dihydroxy fatty acids 12,13-DiHODE via sEH activity^51^. The lipidomic data show that the levels of 9(10)-EpODE, 12(13)-EpODE, and 15(16)-EpODE were significantly upregulated in ibuprofen treated male livers relative to controls (Table 1, Fig. 6A). However, a trend towards reduced 9(10)-EpODE, 12(13)-EpODE, and 15(16)-EpODE levels was observed in ibuprofen treated female livers compared to control groups (Table 1, Fig. 6B).

#### Effect of ibuprofen on CYP-derived oxylipins through linoleic acid (LA) pathway

No difference was observed in the levels of 9,10-DiHODE, 12,13-DiHODE, or 15,16-DiHODE in female livers but they all were reduced in livers from ibuprofen treated male mice relative to controls (Table 1). LA is also metabolized via CYP to epoxy**-**octadecenic acid (EpOME) and is further converted to dihydroxy-octadecenoic acid (DiHOME) by sEH activity^52^. In male livers treated with ibuprofen the level of 12(13)-EpOME was significantly greater than controls, while no change was observed in female livers treated with ibuprofen, showing sex-specific differences (Table 1). 9,10-DiHOME and 12,13-DiHOME diols were decreased in livers from male ibuprofen treated livers. However, no fatty acid diols were significantly altered in livers from female ibuprofen treated mice.

### LOX-derived oxylipins

The LOX enzymes (5-LOX, 8-LOX, 12-LOX and 15-LOX) catalyze the conversion of LA, AA or EPA to hydroperoxy intermediates such as leukotrienes, lipoxins, hepoxillins and HETEs^44,53^. These oxylipins play important functional roles in different cellular processes such as inflammation, cellular proliferation, and intracellular signaling ^53,54^. The LA oxylipin, 9-HODE, can be formed non-enzymatically, or may be formed by the action of LOX or COX^55^. Interestingly, the levels of 9-HODE are downregulated in male livers treated with ibuprofen with no difference in female livers relative to normal controls (Fig. 6C,D). The metabolism of EPA by 8-LOX yields 8-HpEPE which can then be converted to 8-HEPE. The levels of 8-HEPE were found to be elevated in male livers treated with ibuprofen, whereas no difference was observed in female livers treated with ibuprofen compare to control groups (Fig. 6E,F). Similarly, 12-LOX, which plays an important role in tumor angiogenesis, motility, invasion, and metastasis^56^, can synthesize 12-HpEPE from EPA metabolism which can be converted to 12-HEPE. An increase in 12-HEPE levels was observed in ibuprofen-treated male livers (668.2 ± 591.7) relative to control groups (124.8 ± 113.2) (Table 1). No difference in 12-HEPE was observed in female livers.

### COX- derived oxylipins

**Figure 7:**
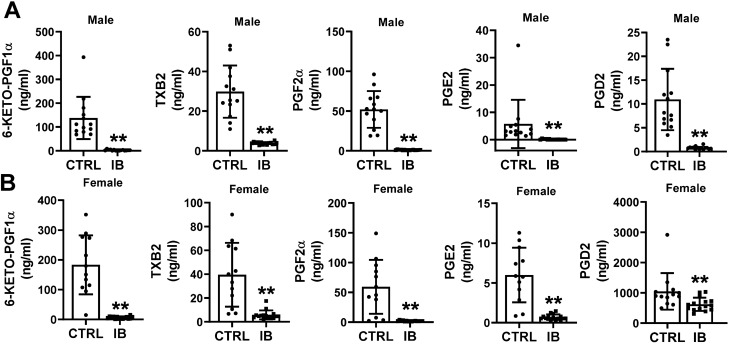
Effect of ibuprofen treatment on COX-derived oxylipins in liver tissue of mice. Boxplots showing the change in the levels of 6-keto-PGF1a, TXB2, PGF2α, PGE2, and PGD2 in (**A**) male liver and (**B**) female liver. Values are mean ± SE; n = 12–15 per group. *p < 0.05.

Prostaglandins derived through the metabolism of AA by enzymatic action of COX enzymes (COX1 and COX2) are known to play major roles in several biological processes including inflammation, vascular tone and platelet aggregation^57,58^. Since ibuprofen is well established to inhibit COX1 and COX2 it was expected that the levels of prostanoids derived from AA metabolism including PGD2 and PGF2α, would be significantly reduced in the livers from both male and female mice treated with ibuprofen relative to their normal controls. (Fig. 7). PGD2, PGE2, PGF2α, 6-keto-PGF1α and TXB2 were all found to be significantly decreased in livers from ibuprofen treated male and female mice. These results suggest that the moderate concentrations of ibuprofen used in the mice inhibited liver COX1 and COX2 as expected.

### Other oxylipins altered

**Figure 8:**
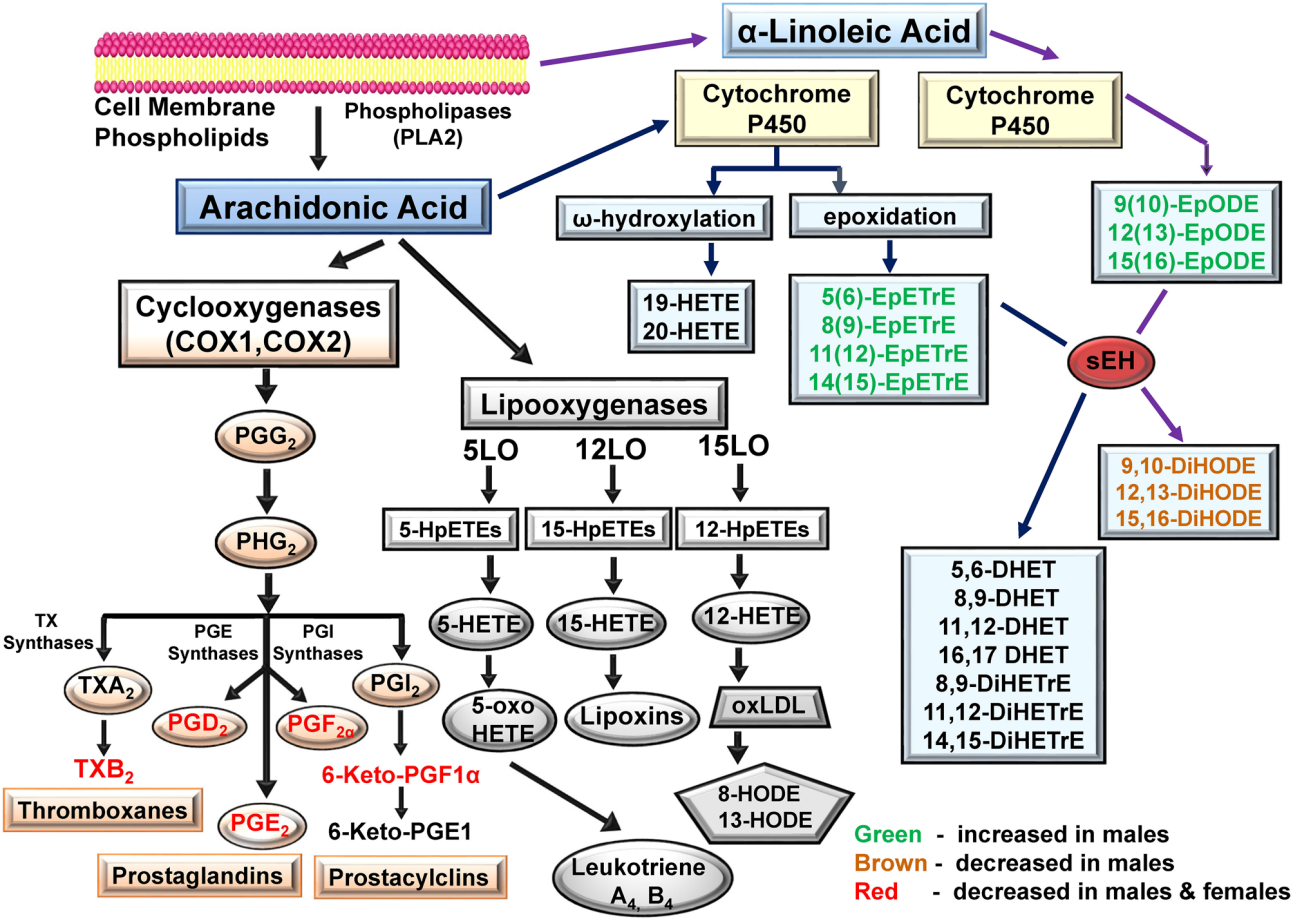
Schematic diagram showing AA and ALA-derived oxylipins that were altered by ibuprofen treatment. Green indicates that oxylipin is increased in males. Red indicates that oxylipin is decreased in both males and females. Brown indicates that oxylipin is decreased in males. Abbreviations: COX1 (Cyclooxegenase-1); COX2 (Cyclooxegenase-2); AA (Arachidonic acid); ALA (α-Linoleic Acid); LA (Linoleic Acid); LO (lipoxygenase); HETE (hydroxyeicosatetraenoic acid); oxo-ETE, oxoeicosatetraenoic acid); HpETE, (hydroperoxyeicosatetraenoic acid); DiHETrE dihydroxyeicosatrienoic acid; EpETrE (epoxy-eicosatrienoic acid); DiHODE (dihydroxy-octadecadienoic acid); EpODE (epoxyoctadecadienoic acid); PG (Prostaglandin).

**Figure 9:**
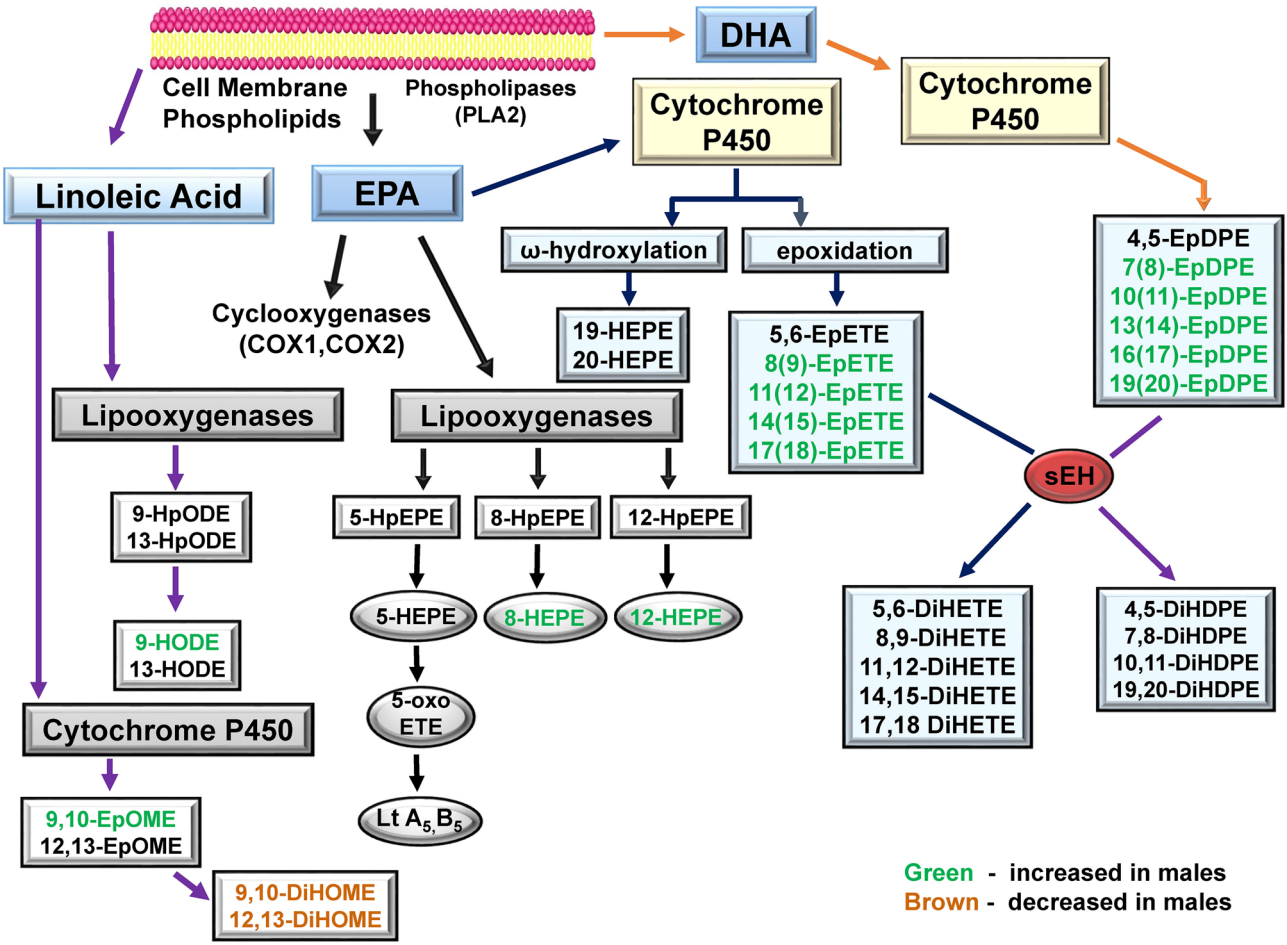
Schematic diagram showing LA, EPA and DHA- derived oxylipins which get altered by ibuprofen treatment. Green indicates that oxylipin is increased in males. Brown indicates that oxylipin is decreased in males. Abbreviations: HEPE (Hydroxyeicosapentaenoic acid); HpEPE (Hydroperoxyeicosapentaenoic acid; oxo-EPE (Oxoeicosapentaenoic acid); DiHETE (Dihydroxyeicosatetraenoic acid); EpETE (Epoxyeicosatetraenoic acid); LA (linoleic acid); EPA (Eicosapentaenoic acid); DHA (Docosahexaenoic acid); DiHDPE (Dihydroxydocosapentaenoic acid); EpDPE (Epoxydocosapentaenoic acid).

A summary of some of the changes in oxylipins observed are shown in Figs. 8 and 9. Figure 8 shows AA and ALA-derived oxylipins that were altered by ibuprofen treatment. Figure 9 shows LA, EPA and DHA- derived oxylipins which were significantly changed by ibuprofen treatment. These diagrams indicate ibuprofen induced more changes to oxylipins in male livers compared to female livers.

### Effect of ibuprofen on CYP450 enzymes

Using the same mass spectrometry data and Scaffold program that was utilized to determine the protein levels of sEH, all data related to CYP enzymes was extracted and analyzed^27^. The results obtained suggest that most liver CYP enzymes were not significantly altered by ibuprofen (Supplemental Fig. 6, Supplemental Table 1). However, CYP4A14, CYP 4A10, CYP 2A5, CYP3A13, CYP2A12, CYP2B9 were increased while CYP2C70, CYP2C39, CYP1A2 were decreased in ibuprofen treated mice compared to vehicle treated mice. A limitation of these results is that proteomics was only done on male mice.

### Sex dependent differences

After a search of the literature to determine the best way to statistically compare sex dependent differences caused by a drug we were surprised that the publications we observed did not do any specialized statistical tests and just compared the results from male animals with female animals. Therefore, to test for differential ibuprofen effects between sexes, we used the sex-ibuprofen interaction effect from a two-factor ANOVA model (see Methods) to test for differential ibuprofen effects between sexes. The script used for the R program to run the analyses is included in the supplemental file. A total of 18 lipids were found to be statistically different when the log of the fold change of the ibuprofen treated group (LogFC.IB) versus the log of the fold change of the control group (logFC.CTRL) for livers from female mice were compared to the LogFC.IB versus logFC.CTRL for livers from male mice (Table 2).

**Table 2:** Male and female liver oxylipins from control and ibuprofen treated mice grouped by sex interaction that showed statistically significant differences.

Lipid	logFC.IB_v_CTR_F	logFC.IB_v_CTR_M	P. Value	adj. P. Val
17(18)-EpETE	− 0.08929	1.44439	0.00004	0.00288
19(20)-EpDPE	− 0.25530	1.13161	0.00012	0.00426
14(15)-EpETE	− 0.08011	1.44210	0.00043	0.00884
11(12)-EpETE	0.18567	1.37971	0.00056	0.00884
8(9)-EpETrE	0.10853	1.37898	0.00063	0.00884
10(11)-EpDPE	0.08053	1.15134	0.00171	0.01583
15(16)-EpODE	− 0.55614	0.75520	0.00180	0.01583
11(12)-EpETrE	− 0.02230	1.46678	0.00181	0.01583
13(14)-EpDPE	0.12494	1.11949	0.00225	0.01748
9(10)-EpODE	− 0.21526	0.94107	0.00298	0.02089
16(17)-EpDPE	0.10111	1.07893	0.00345	0.02195
12(13)-EpODE	− 0.07269	0.94164	0.00418	0.02303
9-HODE	0.05956	− 0.65337	0.00428	0.02303
9-HOTrE	0.21301	− 0.77013	0.00474	0.02370
8(9)-EpETE	− 0.49665	1.92816	0.00703	0.03089
13-HOTrE	0.28959	− 0.84880	0.00750	0.03089
8,9-DiHETrE	0.47225	− 0.26017	0.00845	0.03285
14(15)-EpETrE	− 0.09894	1.61670	0.00892	0.03286

logFC.IB_v_CTR_ < sex > : log2 fold change for IB/CTR for the indicated sex (M, male; F, female). P. Value: Raw p-value from the test that the log fold changes differ from each other. IB, ibuprofen. CTRL, control. Adjusted P values—Benjamini–Hochberg false discovery rate adjusted p-value.

## Discussion

This study analyzed the effects of ibuprofen treatment on oxylipin metabolites. The ibuprofen dose used in this animal study was similar to concentrations used in previous studies^2,59,60^. The dose of 100 mg/kg/day of ibuprofen used in this study would correspond to a moderate daily dose of ibuprofen in humans. We profiled more than 69 oxylipins originating from different precursors including epoxides (EpOME, EpETrE, EpDPE), diols (DiHOME, DiHETE) and hydroxy fatty acids (HETE, HEPE, HODE, DiHODE, HOTrE) that play major regulatory roles in several biological processes ^19,53,61^. Moderate treatment with ibuprofen (100 mg/kg for 7 days) significantly changed oxylipin profiles of 35 oxylipins out of the 69 detected in quantifiable amounts. These oxylipins were derived from different precursors including AA, EPA, DHA and αLA via the three major enzymatic pathways (COX, LOX and CYP). In livers for male mice 36 oxylipins were altered while 5 oxylipins were altered in livers from females. These results suggest that sex specific differences in oxylipin content occurs as a result of ibuprofen treatment.

High dose and long term NSAID treatments are associated with adverse side effects that include cardiac, gastrointestinal, renal and hepatotoxicity^62,63^. Long term administration of aspirin and ibuprofen to rats altered liver ultrastructure and increased the metabolic activity of some CYP450 enzymes^64^. Previous reports from our lab suggest that at physiological concentrations NSAIDs such as diclofenac, naproxen and meclofenamate sodium altered mitochondrial and proteasome function in cardiac tissue^65,66^. Ibuprofen also alters mitochondrial and proteasome function in liver tissue^27^. As expected, ibuprofen (a non-selective COX inhibitor) treatment significantly decreases the levels of prostaglandins (PGE2, PGD2, PGF2a, 6-keto-PGF1a) and TXB2 derived from AA metabolism in both ibuprofen treated male and female mice livers relative to their normal controls, thereby exerting its anti-inflammatory and anti-analgesic effects. It is well acknowledged that COX-derived prostaglandins are key regulators of inflammation, cellular proliferation and intracellular signaling where PGE2 and TXA2 are pro-inflammatory products of this pathway that activate NF-κB, to promote leukocyte infiltration^53,57,67,68^. Thus, pharmacologic inhibition of PGE2 and TXA2 will promote vasodilatory effects. AA acts as a common substrate for the parallel biosynthesis of eicosanoids derived from COX, LOX and CYP pathways. Epoxy lipids can also be stored in the cell membrane and released in response to certain stimuli^69^.

CYPs are a family of enzymes from 18 gene families, some of which encode enzymes related to eicosanoid metabolism while others are predominantly involved in the detoxification process due to xenobiotics and the biosynthesis of other chemical mediators such as steroid hormones, and other products of endogenous metabolism^44,70,71^. The EETs, being the AA products of lipid–metabolizing CYP enzymes and other EpFAs, are reported to play important regulatory roles in several biological functions including vascular tone, mitogenesis, platelet aggregation, and endothelial cell activation^44,72^. EETs derived from AA via the CYP pathway were reported to produce vasodilatory effects through the activation of smooth muscle large-conductance Ca^2+^-activated K + channels and attenuated endothelial inflammatory responses by inhibiting NF-kB activation^61,73^. EET levels are largely regulated by sEH activity which converts active EETs to physiologically inactive DHETs. The levels of different EETs such as 5(6)-EpETrE, 8(9)-EpETrE 11(12)-EpETrE, 14(15)-EpETrE (derived from AA), 9(10)-EpOME, 12(13)-EpOME, 9(10)-EpODE, 12(13)-EpODE (derived from ALA) 8(9)-EpETE, 11(12)-EpETE, 14(15)-EpETE,17(18)-EpETE (derived from EPA), 7(8)-EpDPE, 13(14)-EpDPE and 16(17)-EpDPE (derived from DHA) were significantly increased in ibuprofen treated male livers compared to controls. No significant changes in the levels of EETs in ibuprofen treated female livers were observed even though sEH activity was also enhanced in livers from ibuprofen treated female mice. Interestingly, consistent with our results, in cows exposed to respiratory syncytial virus, the use of ibuprofen increased lymph node epoxides but decreased plasma diols^74^.

Sex differences related to fatty acid metabolism have been previously shown^75–77^*,* but these studies are limited to a few oxylipins or to in vitro or ex vivo studies^75–79^*.* These results have sometimes been conflicting. Our current results strongly suggest that sex related differences with respect to oxylipin changes due to ibuprofen treatment is substantial (Table 2). From a mechanistic point of view it has been suggested that inhibition of COX1 and COX2 by NSAIDs shifts the availability of free AA towards other pathways including the P450 pathway^80,81^. Another possibility is that since ibuprofen is rapidly metabolized in the liver by oxidative metabolism involving multiple cytochrome P450 (CYPs) enzymes (CYP2C9, CYP2C8)^70,71^, the metabolism of ibuprofen itself could be affecting AA metabolism by CYP. While this could partly explain why livers from male mice show altered CYP P450 derived oxylipins it does not explain why livers from female mice do not show similar changes. The expression data from mass spectrometry suggest that at least nine CYPs are altered in ibuprofen treated mice compared to vehicle treated mice. However, the role of these altered CYPs in oxylipin metabolism is not well understood. The elevated EETs may possibly be due to changes in the activity of CYPs associated with the conversion of different lipids to EETs. One of the CYP enzymes that was upregulated in livers from ibuprofen treated mice was CYP4A14. CYP4A14 knockout mice are utilized as a model of 20-HETE overproduction^82^. It could also be that ibuprofen is activating a transcription factor which increase expression of CYP enzymes and sEH as Nrf2 has been shown to significantly alter levels of Cyp2a5 and mEH^83^.

sEH is a target for the treatment of several diseases including hypertension, diabetes, and stroke^40–43^. It has both C-terminal epoxide hydrolase and N-terminal lipid phosphatase activity, and the epoxide hydrolase has a high affinity for epoxides of fatty acids^84^. It is suggested that inhibition of the hydrolase activity of sEH enhances levels of EETs which in turn reduce blood pressure and prevent and resolve inflammatory diseases^85,86^. The higher expression levels and activity of sEH observed in the livers from the ibuprofen treated mice may be important in the liver reducing the elevated levels of EETs. Several sEH inhibitors have been developed for therapeutic applications. Some NSAIDs, such as naproxen and indomethacin, have been associated with higher blood pressure in humans ^87^. In the studies shown in this manuscript, we did not measure the blood pressure of these mice as studies done on normotensive women showed that a high dose of ibuprofen (2400 mg/day) for up to 7 days did not have an effect on blood pressure ^88^. However, inhibition of sEH activity has been shown to reduce blood pressure in an angiotensin II model of hypertension^89^. In general, sEH seems to reduce high blood pressure but does not change normal blood pressure^89^. Since we observed that sEH activity was significantly increased in both males and females (> twofold in females), in future studies we will measure blood pressure in NSAID treated mice to determine if sEH activity changes are associated with blood pressure changes. If it seems to be associated, we will test if inhibition of sEH with inhibitors could prevent these changes.

Future studies on NSAIDs and sEH in blood pressure regulation are also important since studies on Swedish senior primary care patients showed that drug-disease interactions (a drug prescribed for a disease exacerbates an associated disease) occurred in 10% of patients, with changes in hypertension occurring in some, and the most common interactions with other drugs being interactions with NSAIDs^90^. Although some experimental data suggest that oxylipins are involved in blood pressure regulation this aspect of blood pressure modulation is not well understood. Clinical trials with flaxseed supplementation, which contains high amounts of ALA, suggest that oxylipins derived from ALA have positive effects on blood pressure^91^. Studies on animals and humans have also implicated higher levels of 18-carbon or 20- and 22-carbon ω-3 fatty acids in the diet with slowing of some age related changes^92^. Another aspect of increased sEH activity is the potential for ibuprofen to eventually increase pain or prolong pain as inhibition of sEH activity has been shown in several reports to reduce pain^93^. Although ibuprofen works well at relieving pain, using it long term could possibly result in a person becoming dependent on pain relievers since ibuprofen may be inducing sEH activity which causes an underlying pain in that person.

## Conclusion

We performed lipidomic profiling of oxylipins originating from different precursors including epoxides, diols and hydroxy fatty acids. Our main finding is that moderate amounts of ibuprofen for seven days significantly modulated the profiles of lipid mediators in mice liver when compared to control groups. The data suggest that among the three major fatty acid metabolizing pathways in the arachidonic acid cascade (COX, LOX and CYP), oxylipins derived from the COX and CYP pathways are the most altered metabolites and showed ibuprofen-mediated sex-specific differences in male and female mice liver. The data also show that ibuprofen increases sEH activity in livers of both male and female mice suggesting that altered oxylipins may be another mechanism by which ibuprofen can cause side effects. Increased sEH activity is associated with inflammation of the kidney and other organs as well as higher blood pressure and greater pain levels. AA metabolites have been linked to the pathogenesis of certain types of fibrosis^94^. Interestingly, dual COX2/sEH inhibitor has been shown to alleviate experimentally induced pulmonary fibrosis in mice^94^. Overall, oxylipins are likely to be an important group of molecules that can be modified by diet to reduce disease and age-related problems. Understanding differences between males and females will be essential in optimizing how diet and drugs would influence health and aging.

### Limitations of study

Although 69 oxylipins were detected in our study, many more oxylipins exist but their standards are not easily available or unavailable. It is very likely that many other oxylipins are not yet discovered. Also, although we are using state of the art lipidomic techniques^28,29^ all lipidomic techniques utilize extraction methods that favor some types of oxylipins over others^95^. Hence, it is not possible to conclude if more oxylipins from CYP pathways are altered than oxylipins from the COX pathways. Since these studies were done in mice and it has shown that some human and mouse CYPs have similar metabolic properties^96^, it is appealing to suggest that humans will also show similar differences as observed in mice. It has also been suggested that the laboratory mouse model is an indispensable model for exploring human CYP-mediated activities^96^. However, it has been shown that mice have several more putatively functional CYP genes and pseudogenes than humans^97^. Although the mouse results suggest that humans treated with ibuprofen may also show significant differences in CYPs and oxylipins, it is also likely that there are differences in how CYPs and oxylipins are altered in response to ibuprofen treatment in mice and humans.

## Supplementary Information

Supplementary Information 1.

Supplementary Information 2.

## Abbreviations

AA: Arachidonic acid
ALA: α-Linolenic acid
COX: Cyclooxygenase
CYP: Cytochrome P450
DHA: Docosahexaenoic acid
EPA: Eicosapentaenoic acid
CYPe: Cytochrome P450 epoxygenase
CYPh: Cytochrome P450 hydroxylase
DiHDPE: Dihydroxy-docosapentaenoic acid
DiHETrE: Dihydroxy-eicosatrienoic acid
DiHOME: Dihydroxy-octadecenoic acid
EPA: Eicosapentaenoic acid
EpETrE: Epoxy-eicosatrienoic acid
EpOME: Epoxy-octadecenoic acid
GLA: Gamma-linolenic acid
HDoHE: Hydroxy-docosahexaenoic acid
HEPE: Hydroxy-eicosapentaenoic acid
HETE: Hydroxy-eicosatetraenoic acid
HETrE: Hydroxy-eicosatrienoic acid
HHTrE: Hydroxy-heptadecatrienoic acid
HODE: Hydroxy-octadecadienoic acid
HOTrE: Hydroxy-octadecatrienoic acid
LA: Linoleic acid
LOX: Lipoxygenase
mEH: Microsomal epoxide hydrolase
sEH: Soluble epoxide hydrolase
