# Supplementary material for: Ibuprofen alters epoxide hydrolase activity and epoxy-oxylipin metabolites associated with different metabolic pathways in murine livers
Source: Sci Rep. 2021 Mar 29;11:7042. doi: 10.1038/s41598-021-86284-1 (PMC8007717; doi:10.1038/s41598-021-86284-1)
Supplement: Supplementary file 2 — Supplementary Information 2. [file 41598_2021_86284_MOESM2_ESM.pdf]

Supplementary Data

Supplemental Text - R program values

Supplemental Figure 1. Mass spectrometry data for mouse liver sEH. The primary sequence of sEH showing amino acids detected by mass spectrometry that were quantified are shown in yellow. In the primary sequence, modified amino acids (tandem mass tag (TMT) labeled amino acids, etc.) are highlighted in green. Lower figures show a sample spectrum of a sEH peptide (NLPENFSISQIFSQAM), and the raw quantification data for that peptide. The raw quantification data shows that the TMT-126,127N,127C,128N,128C (placebo treated) and TMT-129N,129C,130N,130C,131 (ibuprofen treated livers) relative intensity data. Each TMT label represents liver sample from one animal.

Supplemental Figure 2. Mass spectrometry data for mouse liver mEH. The primary sequence of sEH showing amino acids detected by mass spectrometry are shown in yellow. In the primary sequence, modified amino acids (tandem mass tag (TMT) labeled amino acids, etc) are highlighted in green. Lower figures show a sample spectrum of a mEH peptide (VFVPTGYSAFPSEILHAPE), and the raw quantification data for that peptide. The raw quantification data shows that the TMT-126,127N,127C,128N,128C (placebo treated) and TMT-129N,129C,130N,130C,131 (ibuprofen treated livers) relative intensity data. Each TMT label represents liver sample from one animal.

Supplemental Figure 3. Effect of ibuprofen treatment on CYP-derived oxylipins through the arachidonic acid pathway in (A) male and (B) female liver tissue of mice. Values are mean ± SE; n =12-15 per group. $^*p < 0.05$.

Supplemental Figure 4. Ponceau stained membranes of male and female liver samples used for Western blotting analysis.

Supplemental Figure 5. Blots of the total Western blotting strips used for quantification of sEH and mEH levels. The entire strips are shown and the bands that were used for quantification are shown with a red arrow.

Supplemental Figure 6. Characterization of Cyp4A10 and Cyp4A14 expression in control and ibuprofen treated mouse livers. (A) Abundance of Cyp4A10 in ibuprofen treated livers relative to control as determined by TMT mass spectrometry (n=5). (B) Abundance of Cyp4A14 in ibuprofen treated livers relative to control as determined by TMT mass spectrometry (n=5).

R Session Information

```
## R version 4.0.2 (2020-06-22)
## Platform: x86_64-pc-linux-gnu (64-bit)
## Running under: CentOS Linux 7 (Core)
##
## Matrix products: default
## BLAS:   /usr/local/lib64/R/lib/libRblas.so
## LAPACK: /usr/local/lib64/R/lib/libRlapack.so
##
## locale:
## [1] C
##
## attached base packages:
## [1] stats     graphics  grDevices datasets  utils     methods   base
##
## other attached packages:
## [1] kableExtra_1.2.1 knitr_1.29       emmeans_1.5.0    limma_3.44.3
## [5] readxl_1.3.1     dplyr_1.0.2
##
## loaded via a namespace (and not attached):
##  [1] Rcpp_1.0.5          plyr_1.8.6          highr_0.8
##  [4] cellranger_1.1.0    pillar_1.4.6        compiler_4.0.2
##  [7] BiocManager_1.30.10 tools_4.0.2         digest_0.6.25
## [10] viridisLite_0.3.0   evaluate_0.14       lifecycle_0.2.0
## [13] tibble_3.0.3        pkgconfig_2.0.3     rlang_0.4.7
## [16] rstudioapi_0.11     yaml_2.2.1          mvtnorm_1.1-1
## [19] xfun_0.17           stringr_1.4.0       httr_1.4.2
## [22] xml2_1.3.2          generics_0.0.2      vctrs_0.3.4
## [25] webshot_0.5.2       tidyselect_1.1.0    glue_1.4.2
## [28] R6_2.4.1            rmarkdown_2.3       purrr_0.3.4
## [31] magrittr_1.5        scales_1.1.1        ellipsis_0.3.1
## [34] htmltools_0.5.0     rvest_0.3.6         xtable_1.8-4
## [37] colorspace_1.4-1    renv_0.12.0         stringi_1.5.3
## [40] estimability_1.3    munsell_0.5.0       crayon_1.3.4
```

HYES_MOUSE (100%) 62,516.8 Da
Bifunctional epoxide hydrolase 2 OS=Mus musculus OX=10090 GN=Ephx2 PE=1 SV=2

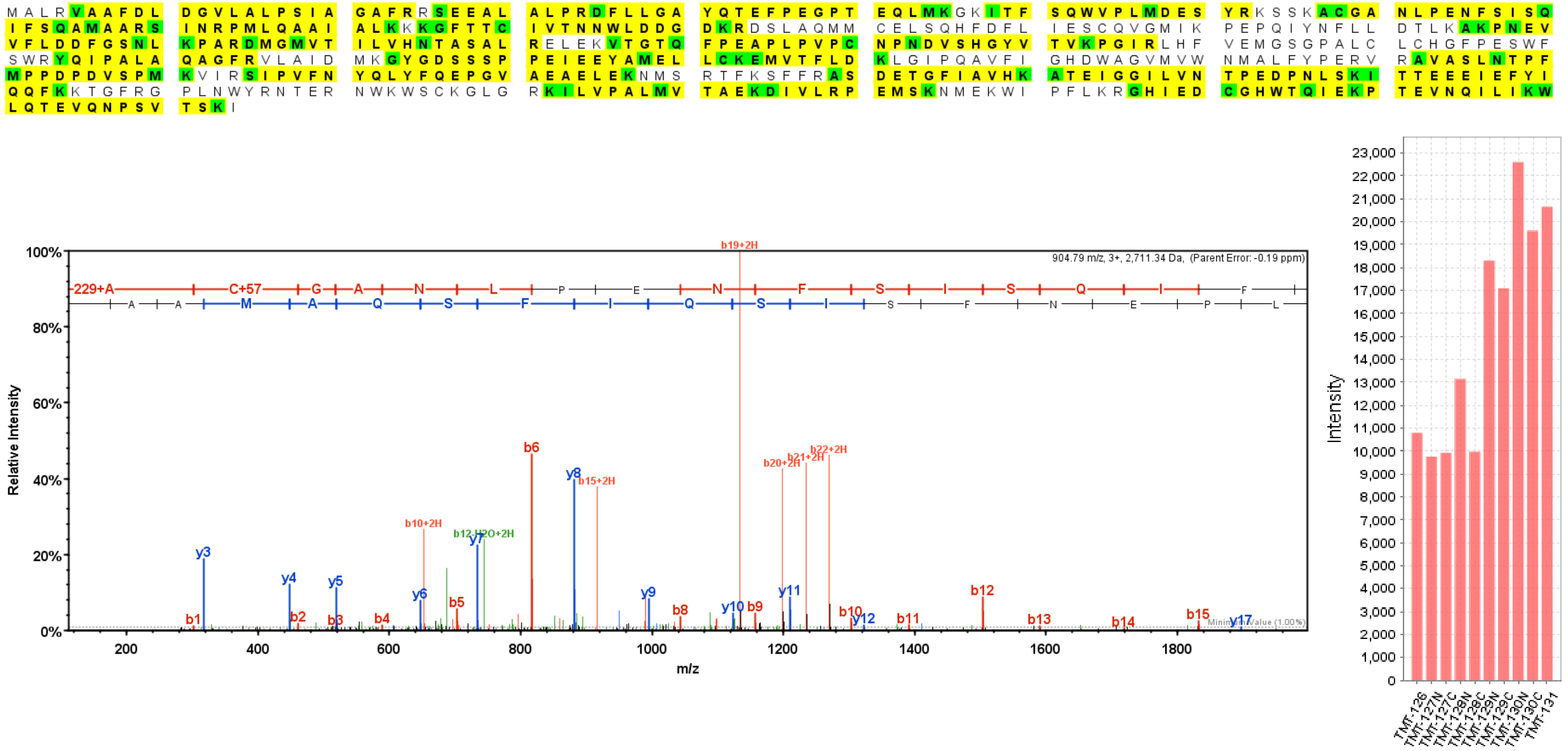

Supplemental Figure 1.

HYEP_MOUSE (100%) 52,578.7 Da
Epoxide hydrolase 1 OS=Mus musculus OX=10090 GN=Ephx1 PE=1 SV=2

```
MWLELILASV LGFVIYWFVS RDKEETLPLE DGWWGPGSKP SAKEDESIRP FKVETSDEEI KDLHQRIDRF RASPPLEGSR FHYGFNSSYL
KKVVSFWRNE FDWRKQVEIL NQYPHFKTKI EGLDIHFIHV KPPQLPSGRT PKPLLMVHGW PGSFYEFYKI IPLLTDPKTH GLSDEHVFEV
ICPSIPGYGF SEASSKKGLN SVATARIFYK LMSRLGFQKF YIQGGDWGSL ICTNIAQMVP NHVKGLHLNM SFISRNIYSL TPLLGQRFGR
FLGYTEKDLE LLYPFKEKVF YNIMRESGYL HIQATKPDTV GCALNDSPVG LAAYILEKFS TWTKSEYREL EDGGLERKFS LEDLLTNIMI
YWTTGTIVSS QRFYKENLGQ GVMVHRHEGM KVFVPTGYSA FPSEILHAPE KWVKVKYPKL ISYSYMERGG HFAAFEEPKL LAQDIRKFVS
LAELQ
```

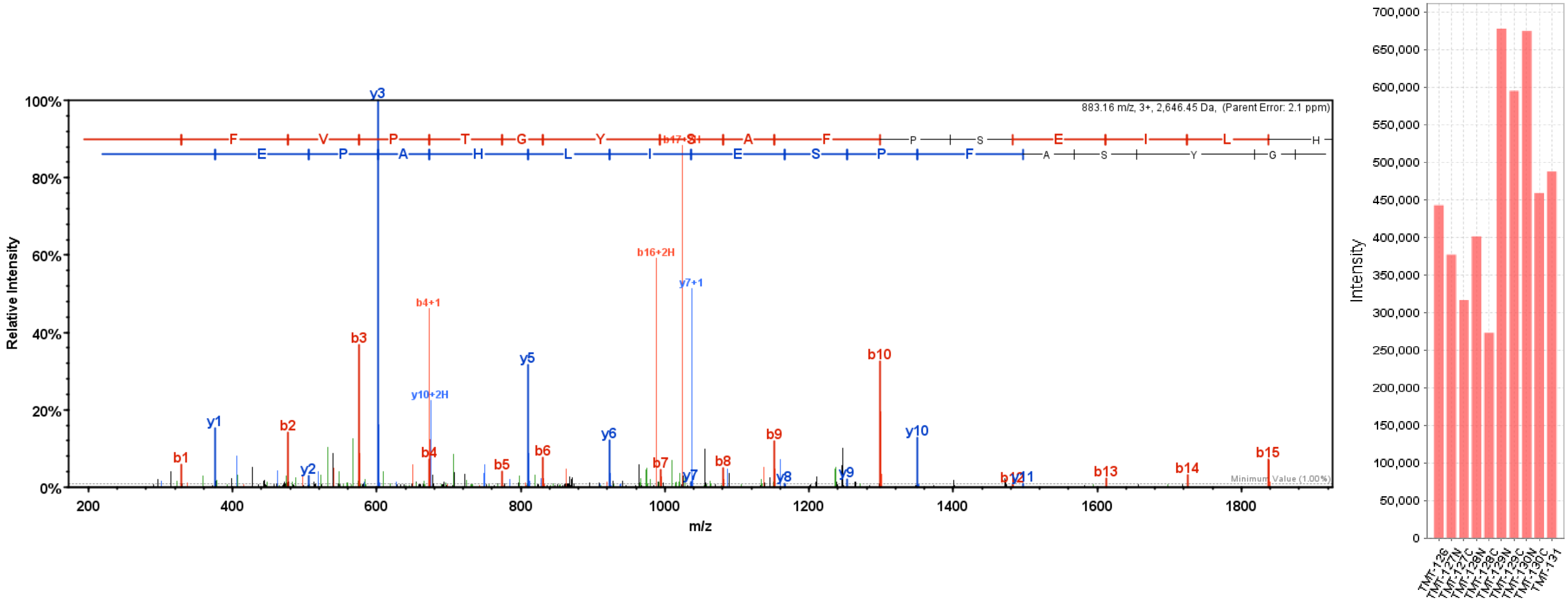

Supplemental Figure 2.

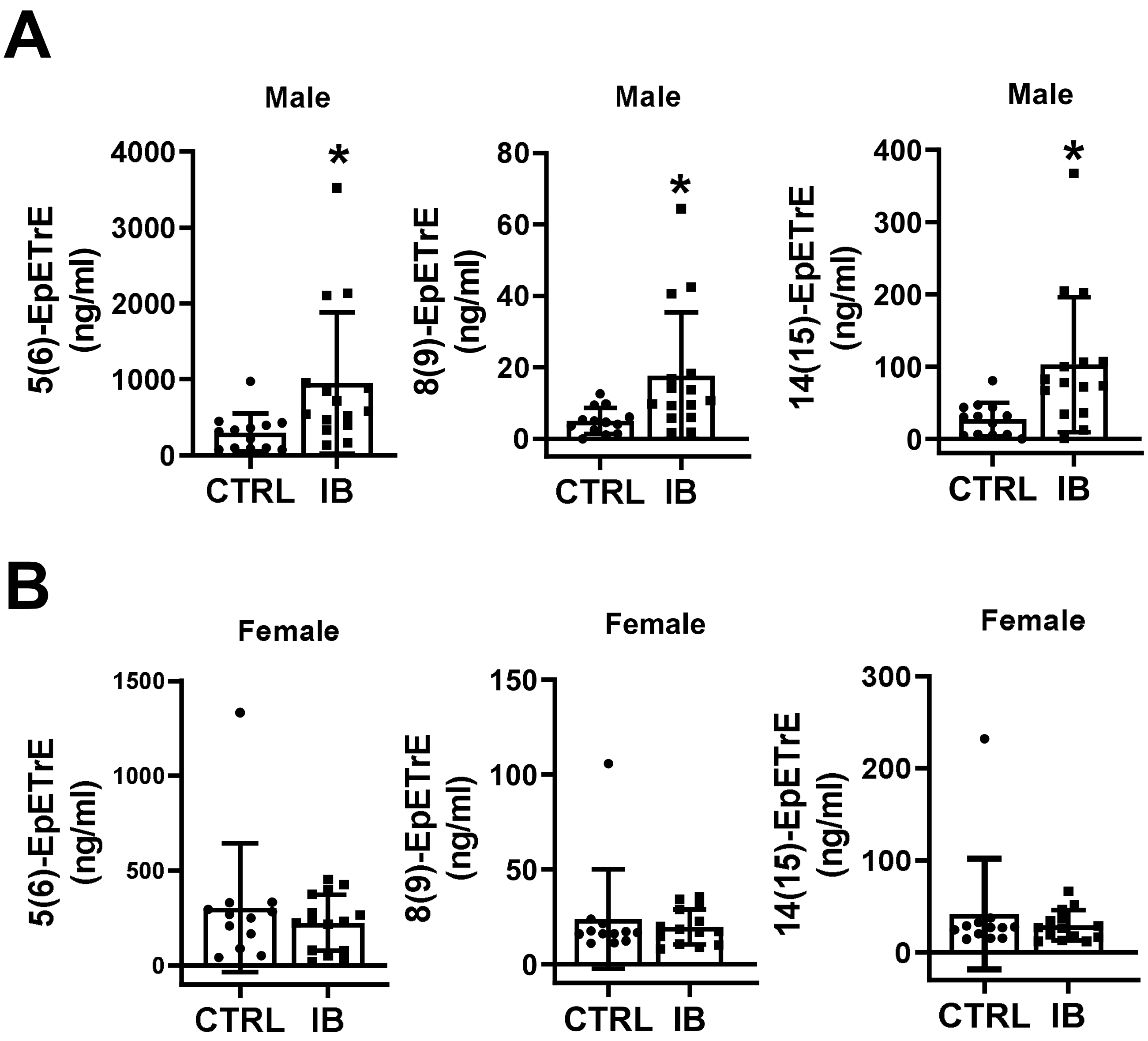

Supplemental Figure 3

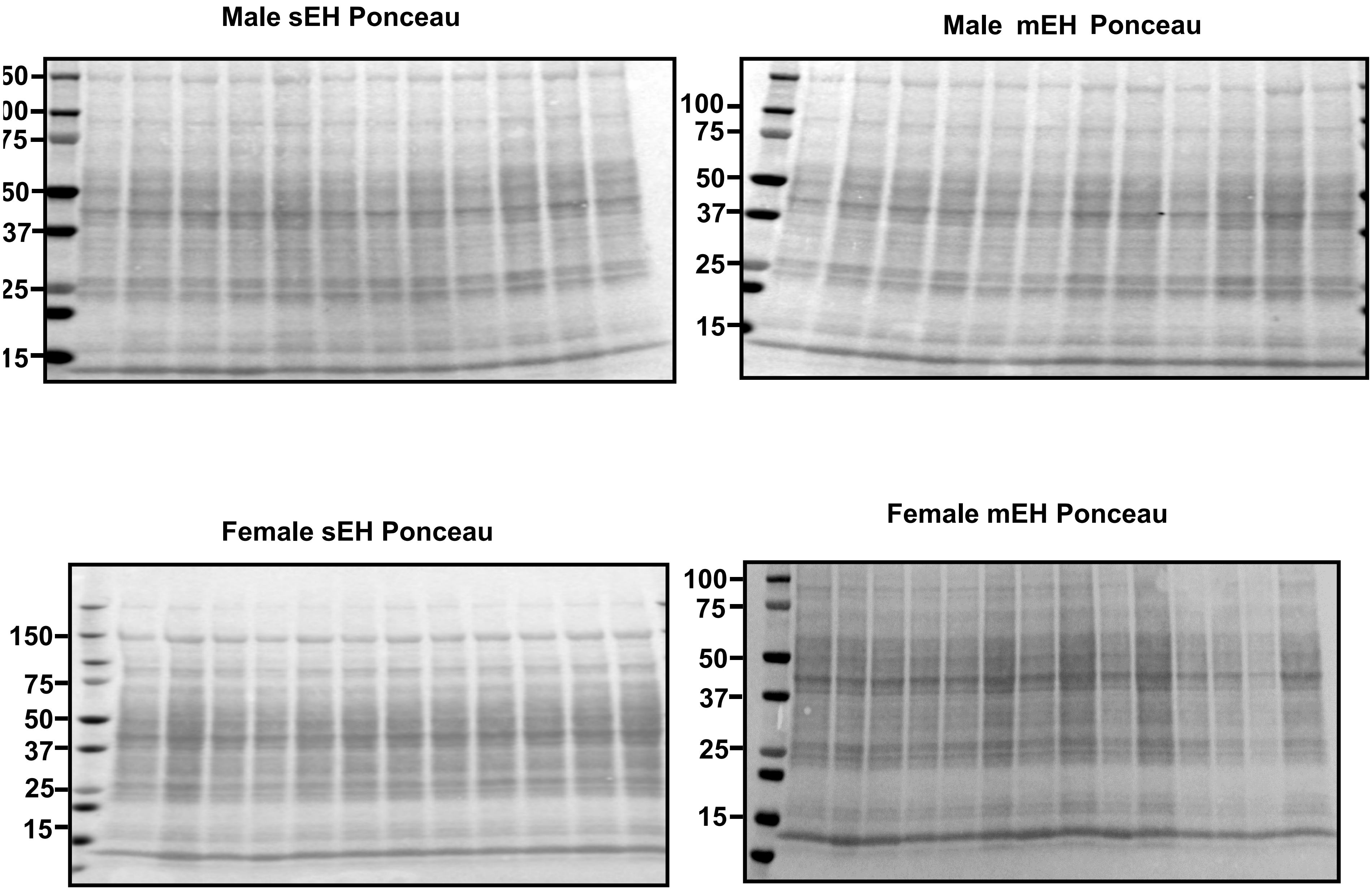

Supplemental Figure 4.

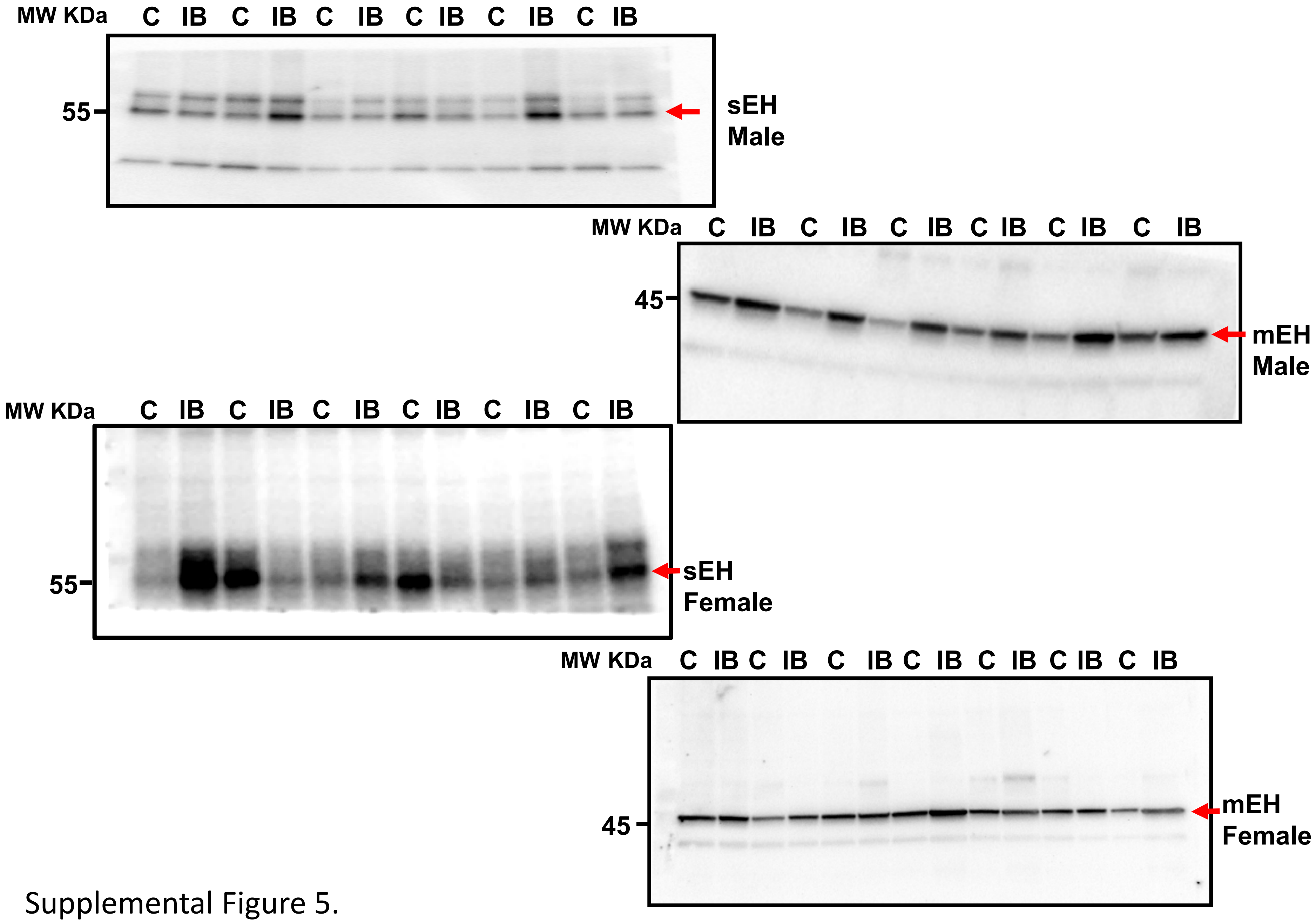

Supplemental Figure 5.

A

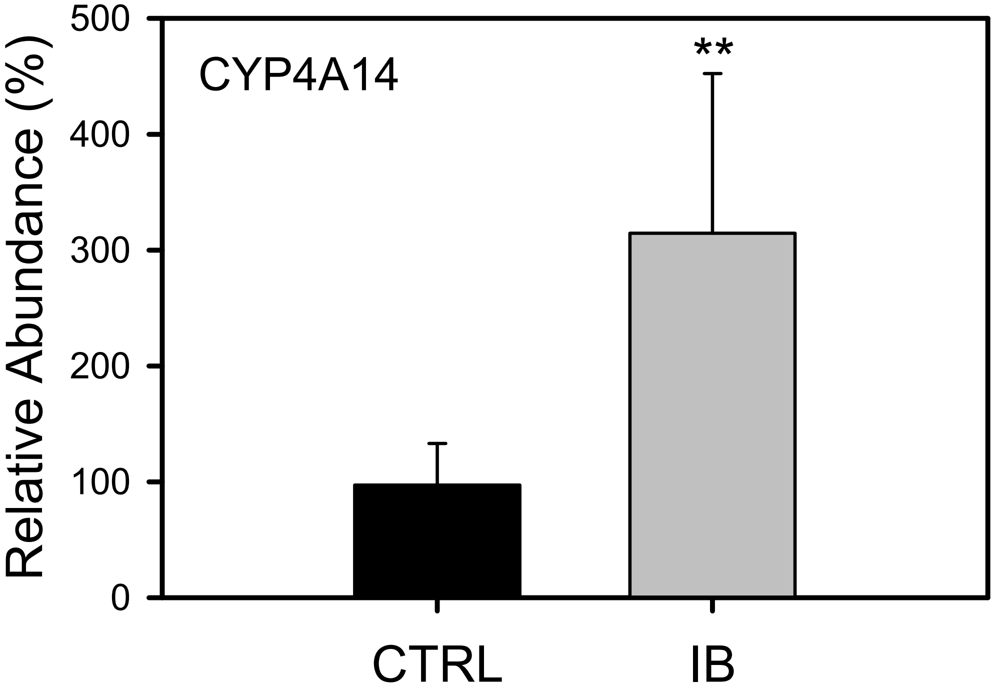

B

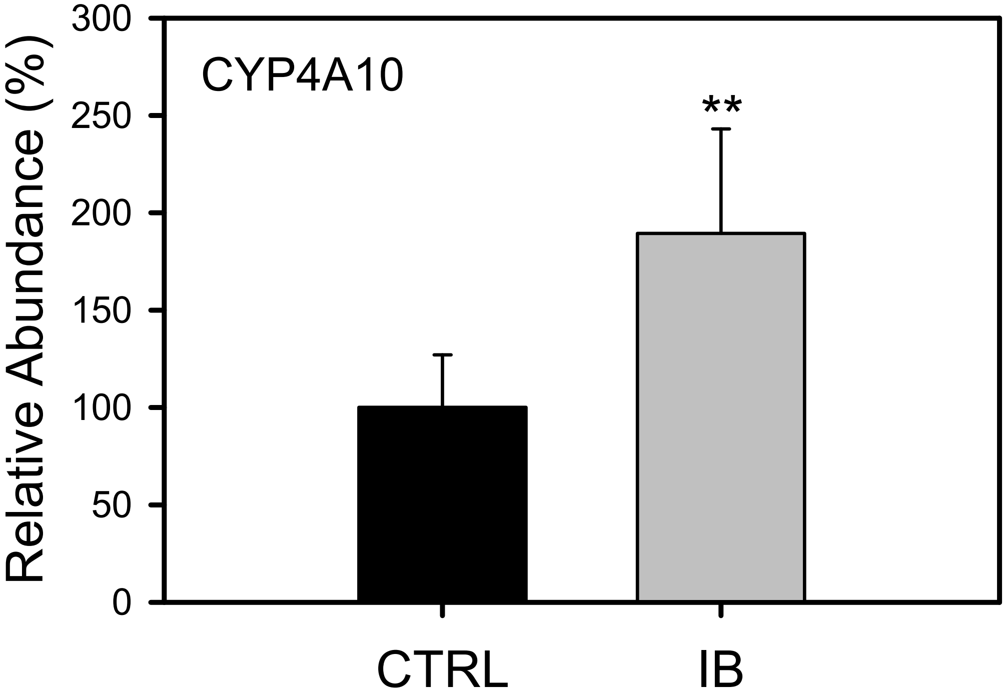

Supplemental Figure 6.

Supplemental Table 1. Expression of CYPs in livers from Ibuprofen and Vehicle treated mice.

CYP 450	Molecular Weight	Permutation Test (p-value) Benjamini-Hochberg (< 0.00393)	Expression Intensity. Ibuprofen/Vehicle Control
CYP4A14	59 kDa	< 0.0001*	3.23
CYP4A10	57 kDa	< 0.0001*	1.90
CYP2A5	57 kDa	< 0.0001*	1.20
CYP2C70	56 kDa	< 0.0001*	0.86
CYP2C39	56 kDa	0.00039*	0.79
CYP2B9	56 kDa	0.0005*	1.14
CYP1A2	58 kDa	0.001*	0.94
CYP3A13	57 kDa	0.001*	1.2
CYP2A12	56 kDa	0.002*	1.10
CYP2C23	56 kDa	0.012	0.92
CYP2E1	57 kDa	0.023	1.04
CYP3A41A	58 kDa	0.12	0.79
CYP2B10	56 kDa	0.19	1.06
CYP4V2	61 kDa	0.19	0.94
CYP3A44	58 kDa	0.22	0.96
CYP2C29	56 kDa	0.23	0.96
CYP20A1	52 kDa	0.25	1.02
CYP2d26	57 kDa	0.29	1.02
CYP2C54	56 kDa	0.3	0.86
CYP2J5	58 kDa	0.34	1.04
CYP2C69	56 kDa	0.34	0.98
CYP4F37	63 kDa	0.41	1.14
CYP2G1	57 kDa	0.51	1.02
CYP4B1	59 kDa	0.52	1.08
CYP3A16	58 kDa	0.69	1.27
CYP2D9	57 kDa	0.72	0.96
CYP2F2	56 kDa	0.74	1.00-
CYP2J7	58 kDa	0.75	1.02
CYP3A25	58 kDa	0.87	1.0
CYP2D22	56 kDa	0.90	0.98
CYP3A11	58 kDa	0.93	0.90
CYP4F15	61 kDa	0.97	0.90

Expression levels was determined by Mass Spectroscopy. * - Statistically significant based upon permutation test (p-value) Benjamini-Hochberg (< 0.00393).
